# Phenotypic Timeline Kinetics, Integrative Networks, and Performance of T- and B-Cell Subsets Associated with Distinct Clinical Outcome of Severe COVID-19 Patients

**DOI:** 10.3390/microorganisms12112272

**Published:** 2024-11-09

**Authors:** Gabriela de Oliveira, Ismael Artur Costa-Rocha, Nani Oliveira-Carvalho, Tâmilla Mayane Alves Fidelis dos Santos, Ana Carolina Campi-Azevedo, Vanessa Peruhype-Magalhães, Vitor Hugo Simões Miranda, Roberta Oliveira Prado, Agnes Antônia Sampaio Pereira, Clarice Carvalho Alves, Joaquim Pedro Brito-de-Sousa, Laise Rodrigues Reis, Christiane Costa-Pereira, Camila Pacheco Silveira Martins da Mata, Vanessa Egídio Silveira Almeida, Liliane Martins dos Santos, Gregório Guilherme Almeida, Lis Ribeiro do Valle Antonelli, Jordana Grazziela Coelho-dos-Reis, Andréa Teixeira-Carvalho, Olindo Assis Martins-Filho

**Affiliations:** 1 Grupo Integrado de Pesquisas em Biomarcadores, Instituto René Rachou, Fundação Oswaldo Cruz-FIOCRUZ-Minas, Belo Horizonte 30190-002, MG, Brazil; gabriela.oliveira@fiocruz.br (G.d.O.); ismael.rocha@fiocruz.br (I.A.C.-R.); nanicarvalhoss@gmail.com (N.O.-C.); tamillamayane@gmail.com (T.M.A.F.d.S.); ana.azevedo@fiocruz.br (A.C.C.-A.); vanessa.pascoal@fiocruz.br (V.P.-M.); vitor.miranda@fiocruz.br (V.H.S.M.); roberta.prado@fiocruz.br (R.O.P.); agnes.pereira@fiocruz.br (A.A.S.P.); clarice.alves@fiocruz.br (C.C.A.); laise_reis@hotmail.com (L.R.R.); christiane.pereira@fiocruz.br (C.C.-P.); liliane.santos@fiocruz.br (L.M.d.S.); reisjordana@gmail.com (J.G.C.-d.-R.); andrea.teixeira@fiocruz.br (A.T.-C.); 2 Departamento de Imunologia e Parasitologia, Universidade Federal de Uberlândia, Uberlândia 38408-100, MG, Brazil; jdesousa@aluno.fiocruz.br; 3 Hospital Risoleta Tolentino Neves (HRTN), Belo Horizonte 31744-012, MG, Brazil; cpsmatins@yahoo.com.br (C.P.S.M.d.M.); vanessa.almeida@hrtn.fundep.ufmg.br (V.E.S.A.); 4 Biologia e Imunologia de Doenças Infecciosas e Parasitárias, Instituto René Rachou, Fundação Oswaldo Cruz-FIOCRUZ-Minas, Belo Horizonte 30190-002, MG, Brazil; vet.greg@gmail.com (G.G.A.); lis.antonelli@fiocruz.br (L.R.d.V.A.); 5 Departamento de Microbiologia, Instituto de Ciências Biológicas, Universidade Federal de Minas Gerais, Belo Horizonte 31270-901, MG, Brazil

**Keywords:** COVID-19, disease outcome, cellular memory, activation, exhaustion

## Abstract

The present study aimed to evaluate the kinetics of the phenotypic profile and integrative networks of T/B-cells in severe COVID-19 patients, categorized according to disease outcome, during the circulation of the B.1.1.28 and B.1.1.33 SARS-CoV-2 strains in Brazil. Peripheral blood obtained at distinct time points (baseline/D0; D7; D14-28) was used for ex vivo flow cytometry immunophenotyping. The data demonstrated a decrease at D0 in the frequency of CD3^+^ T-cells and CD4^+^ T-cells and an increase in B-cells with mixed activation/exhaustion profiles. Higher changes in B-cell and CD4^+^ T-cells at D7 were associated with discharge/death outcomes, respectively. Regardless of the lower T/B-cell connectivity at D0, distinct profiles from D7/D14-28 revealed that, while discharge was associated with increasing connectivity for B-cells, CD4^+^ and CD8^+^ T-cells death was related to increased connectivity involving B-cells, but with lower connections mediated by CD4^+^ T-cells. The CD4^+^CD38^+^ and CD8^+^CD69^+^ subsets accurately classified COVID-19 vs. healthy controls throughout the kinetic analysis. Binary logistic regression identified CD4^+^CD107a^+^, CD4^+^T-bet^+^, CD8^+^CD69^+^, and CD8^+^T-bet^+^ at D0 and CD4^+^CD45RO^+^CD27^+^ at D7 as subsets associated with disease outcomes. Results showed that distinct phenotypic timeline kinetics and integrative networks of T/B-cells are associated with COVID-19 outcomes that may subsidize the establishment of applicable biomarkers for clinical/therapeutic monitoring.

## 1. Introduction

COVID-19, a disease caused by the respiratory virus SARS-CoV-2, emerged as an outbreak in 2019 in China, spread rapidly around the world, and since then has caused several consequences for the world population. The high rate of virus transmission associated with the rapid disease progression to severe cases has prompted efforts to establish effective measures for disease management and morbidity control [1,2]. While mild to moderate COVID-19 symptoms can be observed for up to 2 weeks, severe symptoms, usually starting around 5–10 days after infection, can rapidly evolve into acute respiratory syndrome, leading to a death outcome. Symptom progression and disease outcome may vary depending on viral variants and host factors, including pre-existing comorbidities or immunological status impairment during infection or distinct immune responses elicited upon disease onset [3,4,5]. The SARS-CoV-2 infection triggers a hyperinflammatory storm and delayed secretion of type I interferon and directly impacts the host immune response, leading to cellular exhaustion. T-cell exhaustion results in uncontrolled viral expansion within the host that can manifest as critical illness or progression to death. In this context, immune cells are constantly activated and exhausted, progressively losing their effector functions [6,7,8,9,10,11,12,13,14,15]. The activation markers CD69 and CD38, as well as the transcription factor T-bet, are upregulated in CD4^+^ and CD8^+^ T-cells during acute SARS-CoV-2 infection. In this line, the expression of CD38 has been associated with high proliferation and cellular toxicity [12,13]. Moreover, cellular exhaustion markers such as PD-1 and TIM-3 have been associated with disease severity and found at high T-cell frequencies in infected patients [7,15]. As the immune response profiles may contribute to disease progression to a severe clinical status, the study of phenotypic and functional changes in the immune system represents a rational way to search for imbalanced profiles, useful for identifying biomarkers applicable for disease progression monitoring [14]. The expression of activation and inhibitory molecules, as well as the magnitude and persistence of pro-inflammatory/regulatory events, such as overactivation of lymphocyte subsets counterbalanced by mechanisms of cellular exhaustion, anergy, and apoptosis, have already been reported as relevant sets of biomarkers with effective application in clinical practice [12,13,14,15,16]. In this sense, severe lymphopenia with decreased frequencies of CD4^+^ and CD8^+^ T-cells, as well as CD19^+^ B-cells, have been associated with worse disease progression, with reverse upon clinical recovery [17,18,19,20]. Additional studies to address and characterize the early and late changes in the cell phenotypic profile of COVID-19 patients represent a robust and strategic research model for detecting novel putative biomarkers with clinical application for monitoring and predicting disease outcomes and therapeutic response. Aligned with this proposal, the present study intended to evaluate the timeline kinetics of phenotypic features of T- and B-cell subsets in severe COVID-19 patients, categorized according to disease outcome. Our findings bring novel insights for clinical management, describing distinct phenotypic timeline kinetics and integrative networks of T- and B-cells and providing putative biomarkers with outstanding performance to classify COVID-19 patients according to disease outcome.

## 2. Material and Methods

### 2.1. Study Population and Design

This was a non-interventional observational study carried out between September 2020 and February 2021 during the COVID-19 pandemic in Belo Horizonte, Minas Gerais, Brazil, performed during the peak circulation of the B.1.1.28 and B.1.1.33 SARS-CoV-2 strains. A total of 100 participants were enrolled in a non-probabilistic convenience sampling, including severe unvaccinated patients infected with SARS-CoV-2 (COVID-19, n = 87) and a control group composed of non-infected individuals—Healthy Controls (HCs, n = 13). The COVID-19 patients were recruited after admission at Hospital Risoleta Tolentino Neves, Belo Horizonte, Minas Gerais, Brazil. The COVID-19 group comprised 87 with severe acute SARS-CoV-2 infection (51 males and 36 females, with ages ranging from 26 to 92 years old). The inclusion criteria consisted of severe COVID-19 diagnosis, comprising patients from both sexes presenting respiratory distress (respiratory rate ≥ 30/min or oxygen saturation ≤ 93% on room air) who were under mechanical ventilation at nurse care or intensive care units. All COVID-19 patients presented neither positive diagnosis for SARS-CoV-2 infection, confirmed by positive RT-PCR for SARS-CoV-2 targeting the E gene or presenting positive results for Interferon Gamma Release Assay (Covi-FERON ELISA—IFN-gamma, SD Biosensor, Suwon, Republic of Korea). Demographic and clinical data were obtained from medical records at enrollment. The most frequent symptoms included dyspnea, cough, and fever. The most frequent comorbidity was hypertension and diabetes. Exclusion criteria consisted of pregnancy and being aged under 18 years old. Patients were enrolled at hospital admission (D0) upon signature of informed consent obtained from participants or their next of kin before inclusion in the present investigation. Patients were monitored for up to 28 consecutive days. Peripheral blood samples (5 mL in EDTA as anticoagulant) were collected from COVID-19 patients at the following three consecutive time points: at baseline (D0, n = 87), seven days after (D7, n = 37) (to assess early immune response profiles), and at 14–28 days (D14–28, n = 30) after inclusion (to evaluate late immune response events). Their hematological profile was assessed at D0, demonstrating altered parameters in COVID-19 patients, particularly those progressing to death (Table S1). Clinical follow-up records of disease outcome were available for 71 COVID-19 patients and subsidized further categorization into two subgroups according to disease outcome, including Discharge (n = 38) and Death (n = 33), with sample collection occurring at three consecutive time points [(D0, n = 38; D7, n = 14 and D14–28, n = 11) and (D0, n = 33; D7, n = 14 and D14–28, n = 11), respectively]. Data regarding symptom onset at admission were available for 35 COVID-19 patients and allowed for the categorization of patients into two subgroups, referred to as 3–10 days (n = 17) and 11–24 days (n = 18), with sample collection occurring at three consecutive time points [(D0, n = 17; D7, n = 17 and D14–28, n = 9) and (D0, n = 18; D7, n = 17 and D14–28, n = 17), respectively].

The healthy control group comprised 13 subjects (06 males and 07 females, ages ranging from 20 to 38 years old) from Belo Horizonte, Minas Gerais, with no previous history of SARS-CoV-2 infection. To exclude cases of asymptomatic SARS-CoV-2 infection, healthy controls were tested for antibodies to the SARS-CoV-2 spike RBD by chemiluminescent microparticle immunoassay (SARS-CoV-2 Quant Assay; Abbott Laboratories, IL, USA) or by Interferon Gamma Release Assay (Covi-FERON ELISA—IFN-gamma, SD Biosensor, Republic of Korea). Blood samples from healthy controls (5 mL in EDTA as an anticoagulant) were collected at a single time point.

All participants provided written informed consent prior to inclusion. The study was carried out per the Helsinki Declaration and Resolution 466/2012 from the Brazilian National Health Council for research involving human subjects. The study protocol was submitted and approved by the ethics and research committee of the Instituto René Rachou—Fiocruz Minas (CAAE 30846920.7.0000.0008).

### 2.2. Ex Vivo Immunophenotyping of Peripheral Blood Samples by Flow Cytometry

Immunophenotypic analysis of peripheral blood leukocytes by flow cytometry was carried out as recommended by the manufacturer. Briefly, aliquots of whole blood (200 µL) were incubated in three parallel batches with fluorescent-labeled monoclonal antibodies (anti-CD3/BV650/clone#SK7 or PerCP/clone#SP34-2; anti-CD19/BV711/clone#SJ25C1; anti-CD4/PE/clone#SK3; anti-CD8/BV510/clone#SK1 or AF700/clone#RPAT0; anti-CD69/BV510/clone#FN50; anti-CD223/BV421/clone#T47-530; anti-CD107a/BV421/clone#H4A3; anti-CD28/BV711/clone#CD28.2; anti-CD38/PE-Cy-7/clone#HIT2; anti-PD-1/BV650/clone#EH12 or APC/clone#MIH4; anti-T-bet/AF647/clone#4B10; anti-TIM-3/BV786/clone#7D3; anti-CD62L/BV421/clone#DREG-56; anti-CD45RO/BV786/clone#UCHL1 and anti-CD27/APC-Cy7/clone#M-T271) for 30 min at room temperature in the dark. All antibodies were purchased from BD Biosciences, Franklin Lakes, NJ, USA. Next, erythrocytes were lysed by adding 3 mL of FACS Lysing Solution™ (BD Biosciences, USA), followed by incubation for 10 min at room temperature in the dark. Cell suspensions were washed with phosphate-buffered saline supplemented with 0.01% sodium azide (PBS). Intracellular staining was carried out after pre-incubation with permeabilizing buffer (PBS supplemented with 0.5% bovine serum albumin, 0.5% saponin) and the addition of anti-T-bet/APC monoclonal antibody for 30 min at room temperature, in the dark. Stained cells were washed once and resuspended in 250 µL of PBS prior to flow cytometer acquisition. Total leukocytes were run in a BD LSRFortessa™ cell analyzer (BD Biosciences, Franklin Lakes, NJ, USA), until reaching a total of 100,000 lymphocytes per sample. FlowJo™ Software, version 10.8, was used to analyze the flow cytometry data using distinct gating strategies to quantify the frequency of cell subsets, as illustrated by representative flow cytometry charts provided in Figure S1. The frequency of CD69^+^, CD223^+^, CD170a^+^, T-bet^+^, CD28^+^, CD38^+^, PD-1^+^, TIM-3^+^, CD62L^−^, CD27^+^, CD45RO^+^ in CD3^+^, CD4^+^ and CD8^+^ T-cells and CD62L^−^, CD27^+^, PD-1^+^ in CD19^+^ B-cells were reported as a percentage of gated cells. The phenotypic profile of CD27 and CD45RO and expression were assessed in CD4^+^ and CD8^+^ T-cells along with CD27 and IgD in CD19^+^ B-cells to define distinct memory subsets, as previously reported [21,22,23,24]. Additionally, the frequency of each cell population and mean fluorescence intensity (MFI) were obtained for activation, exhaustion, and memory markers, including PD-1^+^, CD62L^−^, CD27^+^, CD45RO^+^, and CD27, within the CD4^+^ and CD8^+^ subsets to investigate data dimensionality reduction employing a Uniform Manifold Approximation and Projection (UMAP).

### 2.3. Statistical Analysis and Graphical Representation

Descriptive statistical analyses were performed, and the data normality test was assessed using the Shapiro–Wilk test. Considering the nonparametric distribution of all data sets, the Mann–Whitney test was chosen for a two-group comparative analysis [COVID-19 (D0) vs. HCs]. Multiple comparisons amongst subgroups of disease outcome and healthy controls (Discharge vs. Death vs. HCs) were performed by a Kruskal–Wallis test, followed by the Dunn’s post-test. In all cases, significance was considered at *p* < 0.05. The timeline kinetics profile of ex vivo phenotypic features was reported as signatures of cell phenotypes. For this analysis, the original results reported as continuous variables were converted into categorical data using the global median of each variable as the cut-off. The Fisher’s Exact test compared the proportion above the global median cut-offs. The timeline kinetics of cell phenotypes according to days of symptoms at admission were analyzed by converting the original results, reported as continuous variables, into Z-score values (Z = (X − x^−^)/standard deviation). A Spearman rank test was used to assess the correlation between phenotypic features of T-cells, B-cells, and T-cell subsets. Significant strong correlations (*p* < 0.05 and “r” scores ≥ |0.67|) were used for the comparative analysis of integrative networks. Receiver Operating Characteristic (ROC) curve analysis was employed to obtain the performance indexes (sensitivity-Se and specificity-Sp) of cell phenotypes along the timeline kinetics. The Area Under the ROC Curve (AUC) was used for global accuracy analysis, considering AUC ≥ 0.8 to be moderate and AUC ≥ 0.9 to be an outstanding performance. The GraphPad Prism software, version 8.0.2 (GraphPad Prism, San Diego, CA, USA) was used for this set of statistical analysis. The odds ratio (OR) for disease outcome was assessed by binary logistic regression analysis of cell phenotypes along the timeline kinetics. The Minitab software, version 17.1 (State College, PA, USA), was used for this set of statistical analyses.

Graphical representation of the datasets was assembled using distinct software. Microsoft Excel software (version 2406) was employed to construct color maps using conditional formatting based on pre-set color keys. Cytoscape software, version 9.0.1 (available at https://cytoscape.org, accessed on 4 November 2023), was used to create integrative cell phenotype network cluster layouts. The R software, version 4.3.1, was employed for data dimensionality reduction employing the UMAP algorithm, utilizing the standard options of the UMAP package, version 0.2.10.0. The raw data of selected markers (PD-1^+^, CD62L^−^, CD27^+^, CD45RO^+^, and CD27) were concatenated, and 8000 events for CD4^+^ and 5000 events for CD8^+^ T-cells were randomly chosen along the timeline kinetics (D0, D7, D14–28) to guarantee comparability of data qualitatively. GraphPad Prism software, version 8.0.2 (GraphPad Prism, San Diego, CA, USA), was used to assemble volcano plots constructed as scattering distribution of the difference in proportion (%) of subjects with results above the global median cut-off in COVID-19 subgroups (Discharge or Death) according to HCs [Δ_(Discharge-HC)_ and Δ_(Death-HC)_] versus significance (−Log10 *p* values). Venn diagram analyses were carried out (https://bioinformatics.psb.ugent.be/webtools/Venn/, accessed on 4 November 2023) to identify common and selective cell phenotype subsets along the timeline kinetics amongst groups.

## 3. Results

### 3.1. Phenotypic Profile of T- and B-Cells in Severe COVID-19 Patients at Baseline (D0)

**Figure 1:**
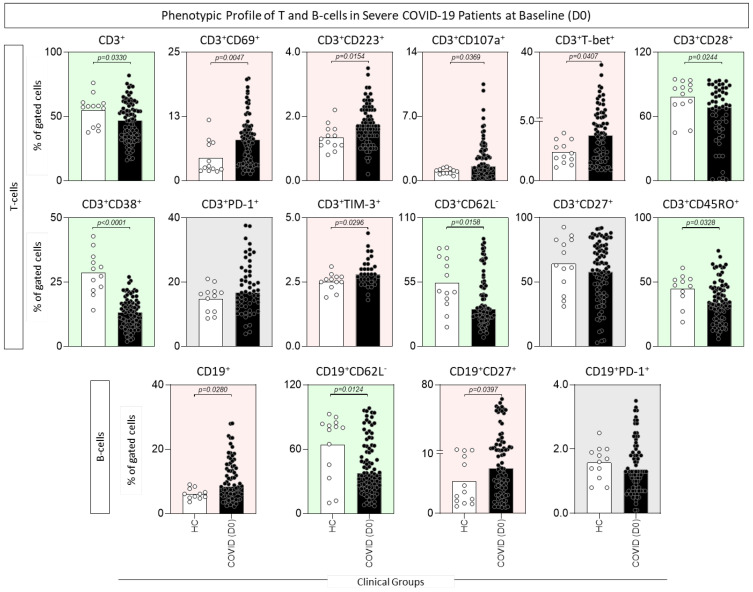
Phenotypic profile of T- and B-cells in severe COVID-19 patients at baseline (D0). Ex vivo phenotypic features of T- and B-cells were assessed in peripheral blood samples collected from COVID-19 patients at D0 (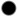, n = 87) and healthy controls (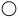, HCs, n = 13). Immunophenotypic staining was carried out as described in Material and Methods. Data are shown as a scattering distribution of individual values over bar charts representing the median percentage (%) of gated cells. Comparative analysis between COVID-19 and HCs was performed by the Mann–Whitney test, and the *p* values for significant differences are provided in the figure. Color backgrounds underscore decreased (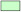), increased (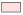), or unaltered (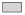) percentages of cell subsets in COVID-19 as compared to HCs.

Aiming to characterize the phenotypic profile of T- and B-cells of COVID-19 patients at D0, ex vivo flow cytometric analysis of peripheral blood samples was carried out, and the results are shown in Figure 1. Data analysis demonstrated that, regardless of the decrease in CD3^+^ T-cells, a mixed profile of activation/exhaustion was observed in the COVID-19 group in comparison to the HC group, as illustrated by increased frequencies of CD69^+^, CD223^+^, CD107a^+^, T-bet^+^, and TIM-3^+^, along with decreased percentages of CD28^+^, CD38^+^, CD62L^−^ and CD45RO^+^ T-cell subsets. Conversely, the increased percentage of B-cells was accompanied by increased frequencies of the CD27^+^ subset and decreased frequencies of CD62L^−^ as compared to the HC group (Figure 1).

**Figure 2:**
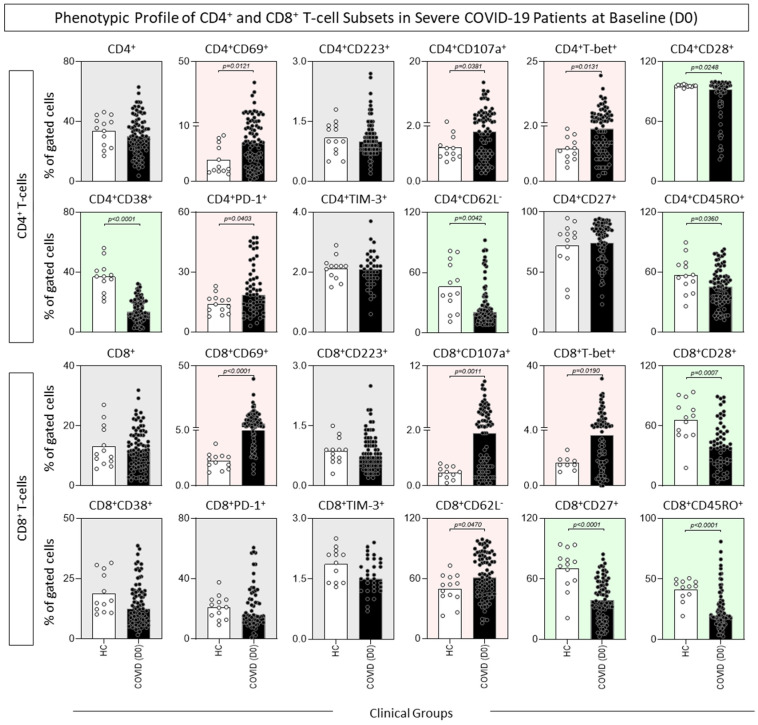
Phenotypic profile of CD4^+^ and CD8^+^ T-cell subsets in severe COVID-19 patients at baseline (D0). Ex vivo phenotypic features of CD4^+^ and CD8^+^ T-cell subsets were assessed in peripheral blood samples collected from COVID-19 patients at D0 (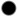, n = 87) and healthy controls (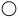, HCs, n = 13). Immunophenotypic staining was carried out as described in Material and Methods. Data are shown as a scattering distribution of individual values over bar charts representing the median percentage (%) of gated cells. Comparative analysis between COVID-19 and HCs was performed by the Mann–Whitney test, and the *p* values for significant differences are provided in the figure. Color backgrounds underscore decreased (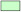), increased (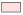), or unaltered (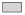) percentages of cell subsets in COVID-19 as compared to HCs.

Descriptive analysis of CD4^+^ and CD8^+^ T-cells subset profiles was also assessed, and the results were reported in Figure 2. Although there was no difference in the frequency of CD4^+^ T-cells, COVID-19 patients presented an increased percentage of CD69^+^, CD107a^+^, T-bet^+^, and PD-1^+^ along with decreased frequencies of CD28^+^, CD38^+^, CD62L^−^ and CD45RO^+^ CD4^+^ T-cells as compared to HCs. Despite no changes in the frequency of CD8^+^ T-cells, increased frequencies of CD69^+^, CD107a^+^, T-bet^+^, and CD62L^−^ and decreased percentages of CD28^+^, CD27^+^, and CD45RO^+^ CD8^+^ T-cells were observed for COVID-19 patients in comparison to HCs (Figure 2).

A complementary analysis of CD4^+^ and CD8^+^ memory T-cell subsets is shown in Figure S2. The results demonstrated that, although no differences were found for the CD4^+^ T-cell subsets, a distinct profile was found for CD8^+^ T-cells with a decreased percentage of Naive (CD45RO^−^CD27^+^) and Central Memory (CD45RO^+^CD27^+^) subsets and an increase in the percentage of early Effector (CD45RO^−^CD27^−^) CD8^+^ T-cells as compared to HCs (Figure S2).

### 3.2. Phenotypic Profile of T- and B-Cells in Severe COVID-19 Patients at Baseline (D0) According to Disease Outcome

**Figure 3:**
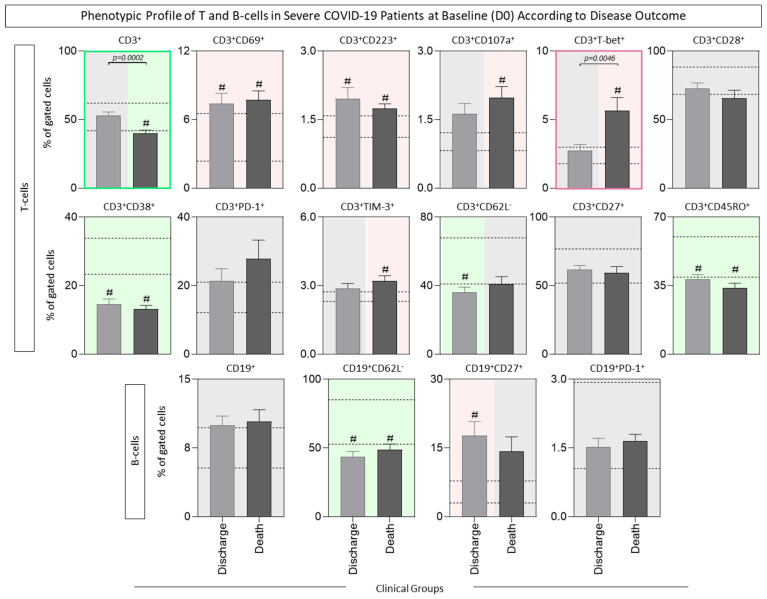
Phenotypic profile of T- and B-cells in severe COVID-19 patients at baseline (D0) according to disease outcome. Ex vivo phenotypic features of T- and B-cells were assessed in peripheral blood samples collected from COVID-19 patients at D0 (n = 71) and further categorized according to disease outcome into Discharge (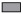, n = 38) or Death (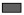, n = 33) groups and compared with the reference range (25th–75th interquartile) of healthy controls (HCs, n = 13, dashed lines). Immunophenotypic staining was carried out as described in Material and Methods. Data are shown as bar charts representing the median percentage (%, 95% CI) of gated cells. Comparative analyses amongst COVID-19 subgroups and HCs were performed using the Kruskal–Wallis test, followed by Dunn’s post-test for multiple comparisons. Significant differences are underscored by # for comparisons with HCs. Significant differences between COVID-19 subgroups were identified by connecting lines, and the *p* values for significant differences are provided in the figure. Color backgrounds underscore decreased (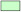), increased (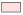), or unaltered (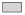) percentages of cell subsets in the COVID-19 subgroups as compared to HCs. Color frames highlight decreased (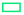) or increased (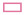) percentages of cell subsets in COVID-19 patients progressing to Death compared to those evolving to Discharge.

The phenotypic profile of T- and B-cells of COVID-19 patients at D0 was further assessed according to Discharge or Death outcomes, and the results are presented in Figure 3. Data analysis demonstrated that several phenotypic features differ in both the Discharge or Death subgroups in comparison to HCs, as observed with the CD69^+^, CD223^+^, CD38^+^, CD62L^−^ and CD45RO^+^ CD3^+^ T-cell subsets, as well as the CD62L^−^ CD19^+^ B-cell subset. Of note is a decreased percentage of CD3^+^ T-cells, in addition to increased frequencies of T-bet^+^ CD3^+^ T-cell subsets, observed in patients progressing to death compared to those evolving to discharge (Figure 3). 

**Figure 4:**
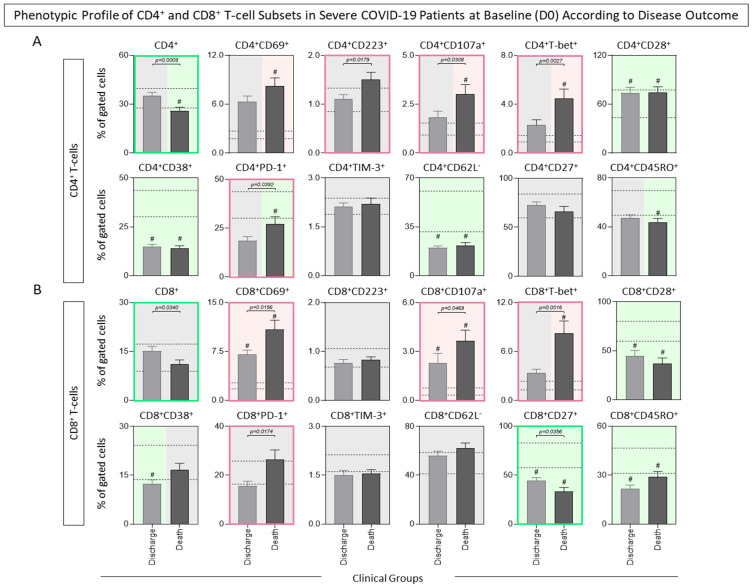
Phenotypic profile of CD4^+^ (**A**) and CD8^+^ (**B**) T-cell subsets in severe COVID-19 patients at baseline (D0) according to disease outcome. Ex vivo phenotypic features of CD4^+^ and CD8^+^ T-cell subsets were assessed in peripheral blood samples collected from COVID-19 patients at D0 (n = 71) and further categorized according to disease outcome into Discharge (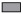, n = 38) or Death (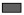, n = 33) groups, then compared with the reference range (25th–75th interquartile) of healthy controls (HCs, n = 13, dashed lines). Immunophenotypic staining was carried out as described in Material and Methods. Data are shown as bar charts representing the median percentage (%, 95% CI) of gated cells. Comparative analyses amongst COVID-19 subgroups and HCs were performed using the Kruskal–Wallis test, followed by Dunn’s post-test for multiple comparisons. Significant differences are underscored by # for comparisons with HCs. Significant differences between COVID-19 subgroups were identified by connecting lines, and the *p* values for significant differences are provided in the figure. Color backgrounds underscore decreased (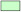), increased (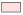), or unaltered (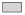) percentages of cell subsets in COVID-19 subgroups as compared to HCs. Color frames highlight decreased (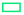) or increased (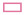) percentages of cell subsets in COVID-19 patients progressing to Death compared to those evolving to Discharge.

Comparative analysis of the phenotypic features of CD4^+^ and CD8^+^ T-cell subsets from COVID-19 subgroups according to disease outcome is shown in Figure 4. Regardless of the disease outcome, decreased frequency of CD28^+^, CD38^+^, and CD62L^−^ CD4^+^ T-cell subsets were observed in both COVID-19 subgroups. Phenotypic profiles associated with clinical progression to death included a reduced frequency of CD4^+^ and an increased percentage of CD223^+^, CD107a^+^, T-bet^+^, and PD-1^+^ subsets (Figure 4A). Analysis of CD8^+^ T-cells showed that, despite the disease outcome, an increased frequency of CD69^+^ and CD107a^+^, along with a decreased percentage of CD28^+^, CD27^+^, and CD45RO^+^ CD8^+^ T-cell subsets, were observed in both COVID-19 subgroups. Notably, lower frequencies of total and CD27^+^ CD8^+^ T-cells, together with increased frequency of CD69^+^, CD107a^+^, T-bet^+^, and PD-1^+^ CD8^+^ T-cell subsets, were observed in COVID-19 patients progressing to death as compared to those evolving to discharge (Figure 4B).

Figure S3 shows the evaluation of memory CD4^+^ and CD8^+^ memory T-cell subsets according to the disease outcome. A decreased frequency of both Naïve and Central Memory CD4^+^ and CD8^+^ T-cells and an increased proportion of early Effector CD4^+^ and CD8^+^ and Effector Memory CD8^+^ T-cells were shown to be decreased in patients progressing to death when compared to the Discharge group (Figure S3). 

Complementary analysis of cell phenotype along the timeline kinetics, according to days of symptom onset at admission, indicated that patients with COVID-19 symptoms between 3 and 10 days after admission to the study presented an overall decrease in cell phenotypes and their subsets (CD3^+^, CD19^+^, CD4^+^ and CD8^+^), with a peak at D7, while patients with symptoms between 11 and 24 days after admission to the study presented a progressive increase in these cell phenotypes mainly at D7 and D14–28 time points_,_ with a peak at D7 for the CD19^+^ phenotype (Figure S4A,B).

### 3.3. Timeline Kinetics Signature and Cell Phenotype Profile in Severe COVID-19 Patients

**Figure 5:**
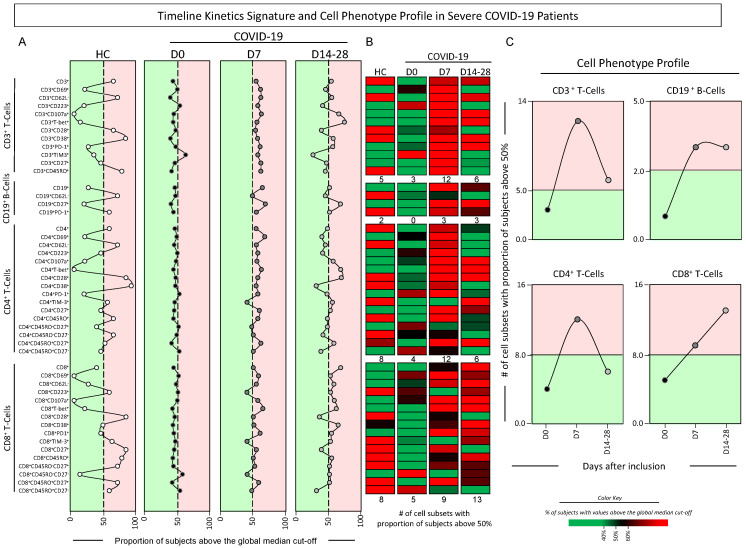
Timeline kinetics signature and cell phenotype profile in severe COVID-19 patients. The timeline kinetic profile of ex vivo phenotypic features of T- and B-cell subsets was assessed in peripheral blood samples collected from COVID-19 patients at distinct time points, including at D0 (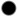, D0, n = 87), seven days (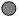, D7, n = 37) and 14–28 days (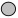, D14–28, n = 30) after inclusion in the study and compared with healthy controls (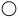, HCs, n = 13). (**A**) Data analyses were carried out by converting the continuous variables measurements (percentage of gated cells) into categorical data reported as the proportion (%) of subjects with results above the global median cut-off (median values of all datasets), as described in Material and Methods. Color backgrounds underscore the cell subsets with the proportion of subjects below (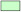) or above (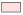) 50% (dashed line). (**B**) Color maps were assembled to underscore the cell subsets with the proportion of subjects below or above 50% according to the color key provided in the figure. (**C**) The number (#) of cell subsets with a proportion above 50% was calculated and data are shown in line charts to illustrate the cell phenotype profile along the days after inclusion. Color backgrounds underscore decreased (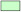) or increased (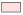) numbers of cell subsets in COVID-19 as compared to the reference values observed in HCs (continuous line).

The kinetic profile of phenotypic features of T- and B-cells was further investigated in COVID-19 patients and reported as signatures and cell phenotype profiles (Figure 5). Data analysis demonstrated that, overall, a decreased proportion of COVID-19 patients with cell subsets above the global median was observed at D0. At D7, an increased proportion of COVID-19 patients above the global median was observed. While a decreased proportion of the CD3^+^ and CD4^+^ phenotypes were observed towards D14–28, a distinct pattern of increased values was found for CD8^+^ T-cells. (Figure 5A). The color map constructs further corroborate these findings and subsidize the analysis of cell phenotype profiles along the timeline kinetics (Figure 5B,C). The results confirmed the lower number of cell subsets altered at D0 (CD3^+^ = 3; CD19^+^ = 0; CD4^+^ = 4; CD8^+^ = 5) as compared to HCs (CD3^+^ = 5; CD19^+^ = 2; CD4^+^ = 8; CD8^+^ = 8). Moreover, data analysis pointed out a pronounced peak of changes in CD3^+^ and CD4^+^ phenotypes at D7 (CD3^+^ = 12; CD4^+^ = 12), with a further decline towards D14-28 (CD3^+^ = 6; CD4^+^ = 6). A distinct pattern was confirmed for B-cells with stable values observed at D7 (CD19^+^ = 3) and D14-28 (CD19^+^ = 3), while, for CD8^+^ T-cells, a further increase was observed at D7 (CD8^+^ = 9) and D14-28 (CD8^+^ = 13) (Figure 5C).

### 3.4. Timeline Kinetics Signature in Severe COVID-19 Patients According to Disease Outcome

**Figure 6:**
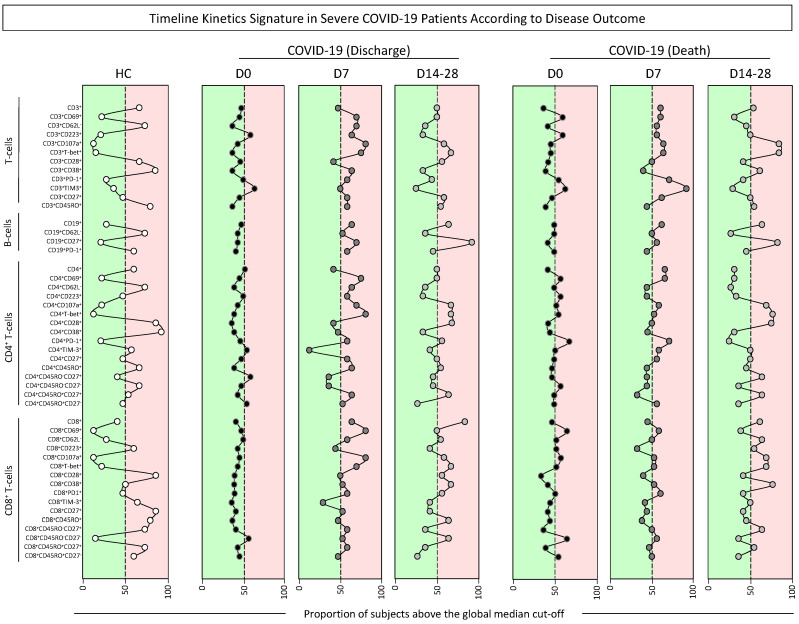
Timeline kinetics signature in severe COVID-19 patients according to disease outcome. The timeline kinetic profile of ex vivo phenotypic features of T- and B-cell subsets was assessed in peripheral blood samples collected from COVID-19 patients further categorized according to disease outcome into Discharge or Death and compared with healthy controls (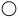, HCs, n = 13). Biological samples were obtained at distinct time points, including at D0 (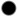, Discharge D0, n = 38; Death D0, n = 33), seven days (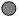, Discharge D7, n = 14; Death D7, n = 14), and 14–28 days (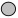, Discharge D14–28, n = 11; Death D14–28, n = 11) after inclusion in the study. Data analyses were carried out by converting the continuous variables measurements (percentage of gated cells) into categorical data reported as the proportion (%) of subjects with results above the global median cut-off (median values of all datasets), as described in Material and Methods. Color backgrounds underscore the cell subsets with the proportion of subjects below (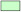) or above (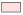) 50% (dashed line).

**Figure 7:**
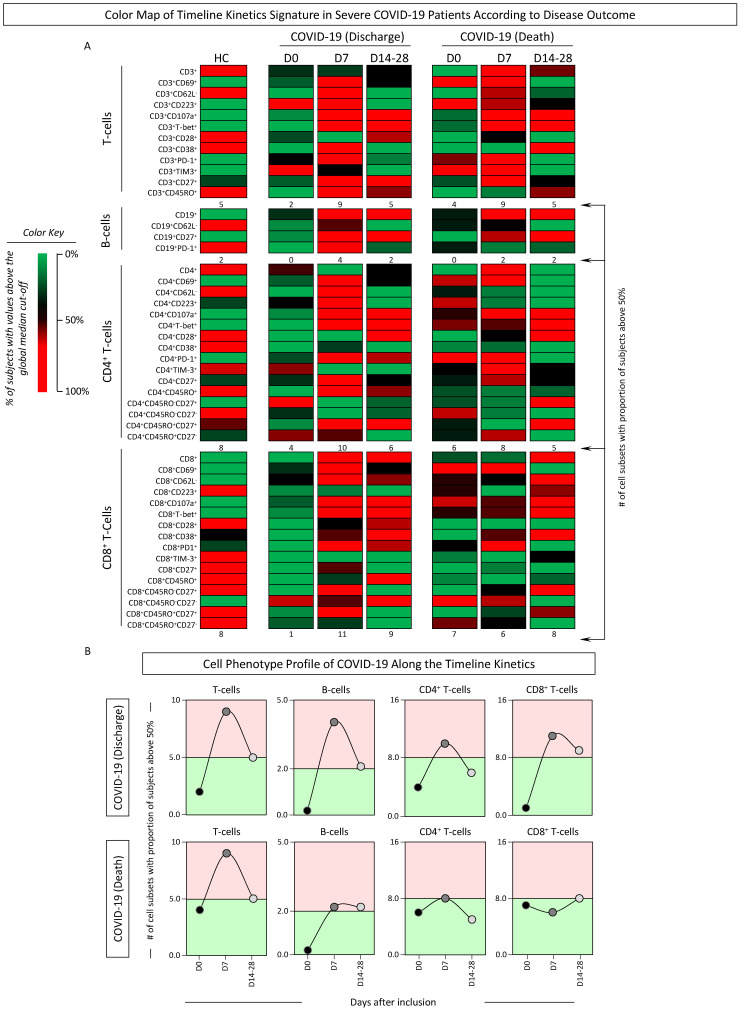
Color map of timeline kinetics signature and cell phenotype profile in severe COVID-19 patients according to disease outcome. The timeline kinetic profile of ex vivo phenotypic features of T- and B-cell subsets was assessed in peripheral blood samples collected from COVID-19 patients further categorized according to disease outcome into Discharge or Death and compared with healthy controls (HCs, n = 13). Biological samples were obtained at distinct time points, including at baseline (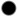, Discharge D0, n = 38; Death D0, n = 33), seven days (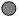, Discharge D7, n = 14; Death D7, n = 14), and 14–28 days (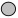, Discharge D14–28, n = 11; Death D14–28, n = 11) after inclusion in the study. Data analyses were carried out by converting the continuous variables measurements (percentage of gated cells) into categorical data to estimate the proportion (%) of subjects with results above the global median cut-off (median values of all datasets), as described in Material and Methods. (**A**) Color maps were assembled to underscore the cell subsets with the proportion of subjects below or above 50% according to the color key provided in the figure. (**B**) The number (#) of cell subsets with a proportion above 50% was calculated, and data were shown in line charts to illustrate the cell phenotype profile along the days after inclusion. Color backgrounds underscore decreased (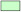) or increased (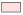) numbers of cell subsets in COVID-19 as compared to the reference values observed in HCs (continuous line).

The kinetic profile of phenotypic features of T- and B-cells was also characterized in COVID-19 patients according to disease outcome, and data were reported as signatures and cell phenotype profiles (Figure 6 and Figure 7). The results showed that, overall, regardless of the disease outcome, a decreased proportion of COVID-19 patients with cell subsets above the global median was observed at D0. Interestingly, at D7, differences between patients evolving to discharge or death became even more evident with an overall increase in the Discharge group, while higher values were observed in patients progressing to death. The most prominent changes in the signature profiles detected towards D14–28 were found for CD4^+^ and CD8^+^ T-cells, with decreased values observed in patients evolving to discharge, contrasting with higher numbers observed for patients progressing to death (Figure 6). Color map data further reinforce these findings and support the analysis of cell phenotype profiles along the timeline kinetics (Figure 7A,B). The results confirmed the overall lower number of cell subsets altered at D0 for patients evolving to discharge (CD3^+^ = 2; CD19^+^ = 0; CD4^+^ = 4; CD8^+^ = 1) and those progressing to death (CD3^+^ = 4; CD19^+^ = 1; CD4^+^ = 6; CD8^+^ = 7) as compared to HCs (CD3^+^ = 5; CD19^+^ = 2; CD4^+^ = 8; CD8^+^ = 8). Data analysis indicated a pronounced peak of changes in CD3^+^ T-cell phenotypes at D7 regardless of the clinical outcome (CD3^+^ = 9 and 9, respectively), with a further decline towards D14-28 (CD3^+^ = 5 and 5, respectively). A distinct pattern was confirmed for B-cells, with increased values observed at D7 and lower values at D14-28 for patients evolving to discharge (CD19^+^ = 4 and 2, respectively), while patients progressing to death frequencies at D7 and D14–28 were the same (CD19^+^ = 2 and 2, respectively) (Figure 7B).

In addition, volcano plots were constructed using the timeline kinetics signature data (Figure 5) for outcome subgroups (Discharge and Death). For the Discharge group, results showed the exclusive cell subsets (bold and underlined) that are decreased at D0 (CD4^+^CD45RO^+^ and CD8^+^CD38^+^), and D14-28 (CD4^+^CD223^+^ and CD4^+^CD45RO^+^CD27^−^), and the exclusive cell subsets (bold and underlined) that are increased at D0 (CD4^+^CD45RO^−^CD27^+^), D7 (CD3^+^TIM-3^+^) and D14-28 (CD3^+^ and CD3^+^CD27^+^). Also, the Death group showed exclusive cell subsets (bold and underlined) decreased in D7 (CD8^+^CD223^+^) and D14–28 (CD4^+^CD223^+^, CD4^+^CD45RO^−^CD27^−^, and CD8^+^CD45RO^+^CD247^−^), as well as exclusive cell subsets (bold and underlined) that are increased at D0 (CD4^+^CD223^+^ and CD8^+^CD45RO^+^CD27^−^), D7 (CD3^+^CD27^+^ and CD8^+^PD-1^+^) and D14-28 (CD4^+^TIM-3^+^, CD4^+^CD45RO^−^CD27^+^, CD8^+^, CD8^+^CD28^+^ and CD8^+^CD38^+^). Moreover, the other cell subsets shown in the graphs in a gray color are present together at more than one time point. The cell subsets that are common between all the time points (D0, D7, and D14–28) are listed in green (decreased) and red (increased) for both outcome subgroups. Together, these results provide distinct patterns for cell phenotypes along the timeline kinetics (D0, D7, D14–28) (Figure S5).

### 3.5. Integrative Network of T- and B-Cell Subsets in Severe COVID-19 Patients at Baseline (D0)

**Figure 8:**
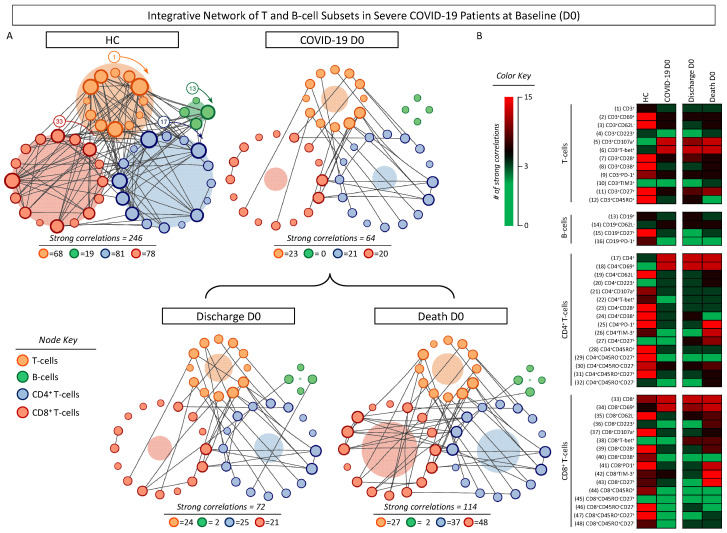
Integrative network of T- and B-cell subsets in severe COVID-19 patients at baseline (D0). Integrative networks were assembled for ex vivo phenotypic features of T- and B-cell subsets from COVID-19 patients at D0 (n = 87), further categorized according to disease outcome into Discharge (n = 38) or Death (n = 33) and compared with healthy controls (HCs, n = 13). Data analyses were carried out by Spearman rank tests, and only significant strong correlations (*p* < 0.05 and “r” scores ≥ |0.67|) were used to construct the integrative networks. (**A**) Cluster layout networks were constructed comprising four groups of cell phenotypes, including CD3^+^ T-cells (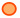), CD19^+^ B-cells (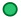), CD4^+^ (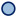) and CD8^+^ (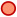) T-cell subsets. Node border thickness is proportional to the number of strong correlations. Connecting edges (black lines) were used to link pairs of cell phenotypes displaying significant correlations. The number of strong correlations observed for each network is provided in the figure and used for comparative analysis between the COVID-19, HCs, and COVID-19 subgroups. The circular background area is proportional to the number of strong correlations of each cluster within the respective network. (**B**) Color map constructs were assembled to illustrate the overall connectivity between cell phenotypes in the COVID-19, HCs, and COVID-19 subgroups. A color key was employed to underscore the cell phenotypes with strong correlations.

Integrative networks were built to evaluate the overall connectivity between distinct T- and B-cell subsets in COVID-19 patients at D0, which were further classified according to disease outcome compared to HCs. The results are presented in Figure 8. Data analysis demonstrated that the COVID-19 group displayed an overall decrease in network connectivity, as evidenced by the small number of strong correlations (n = 64) compared to HCs (n = 246). The analysis of COVID-19 subgroups revealed that patients evolving to discharge presented fewer strong correlations (n = 72) than patients progressing to death (n = 124). Remarkably, differences in connectivity were more pronounced in the CD8^+^ T-cell compartment (Figure 8A). Color map constructs further illustrate these findings, re-emphasizing that COVID-19 elicits a substantial decrease in connectivity between T- and B-cell subsets (Figure 8B).

**Figure 9:**
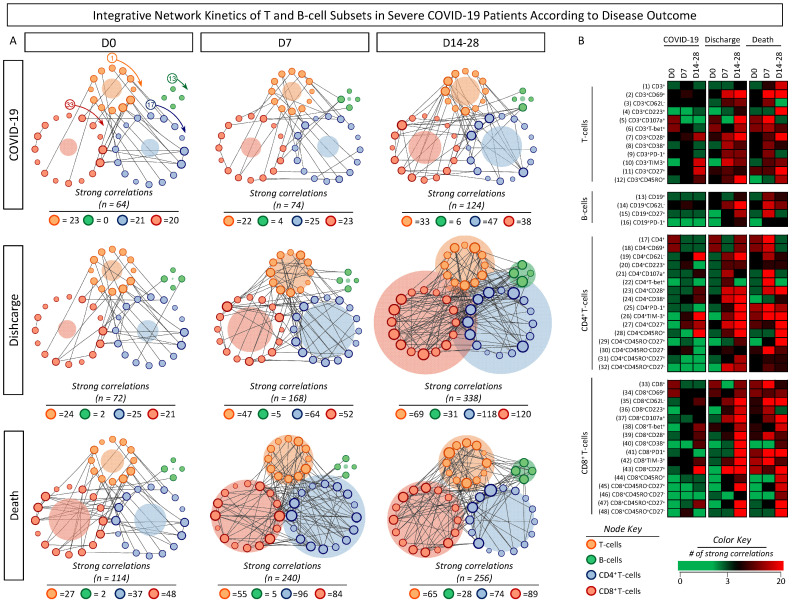
Integrative network kinetics of T- and B-cell subsets in severe COVID-19 patients according to disease outcome. (**A**) Integrative networks were assembled for ex vivo phenotypic features of T- and B-cell subsets from COVID-19 patients at D0 (n = 87), further categorized according to disease outcome into Discharge (n = 38) or Death (n = 33). Biological samples were obtained at distinct time points, including at baseline (Discharge D0, n = 38; Death D0, n = 33), seven days (Discharge D7, n = 14; Death D7, n = 14), and 14–28 days (Discharge D14–28, n = 11; Death D14–28, n = 11) after inclusion in the study. Data analyses were carried out by Spearman rank tests, and only significant strong correlations (*p* < 0.05 and “r” scores ≥ |0.67|) were used to construct the integrative networks. Cluster layout networks were constructed comprising four groups of cell phenotypes, including CD3^+^ T-cells (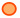), CD19^+^ B-cells (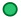), CD4^+^ (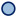), and CD8^+^ (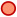) T-cell subsets. Node border thickness is proportional to the number of strong correlations. Connecting edges (black lines) were used to link pairs of cell phenotypes displaying significant correlations. The number of strong correlations observed for each network is provided in the figure and used for comparative analysis between COVID-19 and COVID-19 subgroups according to disease outcome. The circular background area is proportional to the number of strong correlations of each cluster within the respective network. (**B**) Color map constructs were assembled to illustrate the overall connectivity between cell phenotypes in the COVID-19 and COVID-19 subgroups. A color key was employed to underscore the cell phenotypes with strong correlations.

To better understand the kinetics of T- and B-cell subset connectivity, integrative networks were assembled for COVID-19 patients further classified according to disease outcome at distinct time points (D0, D7, D14–28), and the results are presented in Figure 9. Data analysis showed that, in general, COVID-19 subgroups displayed a progressive increase in T- and B-cell subset connectivity (D0 = 76, D7 = 90, D14–28 = 150). The analysis of integrative networks in COVID-19 patients according to the disease outcome showed a pronounced increase from D0 towards D14-28 (Discharge: D0 = 72, D7 = 168, D14–28 = 338 and Death: D0 = 114, D7 = 240, D14–28 = 256). Remarkably, the changes in the network profile from D0 towards D14–28 revealed a distinct pattern between COVID-19 patients evolving to discharge or progressing to death. While patients evolving to discharge presented increasing connectivity at D14–28, patients progressing to death exhibited an early increase in connectivity at D7. Moreover, patients evolving to discharge showed more correlations from D7 to D14–28 involving B-cells (n = 5 → 31), CD4^+^ (n = 64 → 118), and CD8^+^ (n = 52 → 120) T-cells, while patients progressing to death exhibited an increase in the connections involving B-cells (n = 5 → 28) and CD8^+^ T-cells (n = 84 → 89), with an opposite profile being observed for CD4^+^ (n = 96 → 74) T-cells (Figure 9A). These findings were further illustrated by a color map analysis highlighting the notable shift in the network profile dynamic from D7 towards D14–28 with relevant differences between COVID-19 subgroups (Figure 9B).

**Figure 10:**
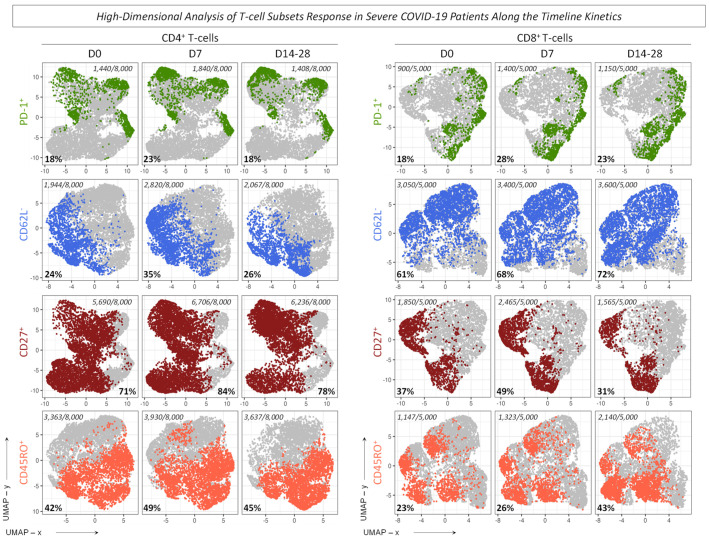
High-dimensional analysis of T-cell subsets in severe COVID-19 patients along the timeline kinetics. Data dimensionality reduction was performed using the UMAP algorithm for selected T-cell subsets. The cell frequency values were extracted from FlowJo software, and the graphical representation shows the timeline kinetics (D0, D7, D14–28) of 8000 events for T CD4^+^ and 5000 events for T CD8^+^ populations. The overall frequency of each cell subset [PD-1^+^ (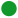), CD62L^−^ (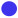), CD27^+^ (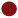), and CD45RO^+^ (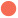)] is illustrated in the dotted clusters and the frequencies are represented in the UMAP x vs. UMAP y axes.

**Figure 11:**
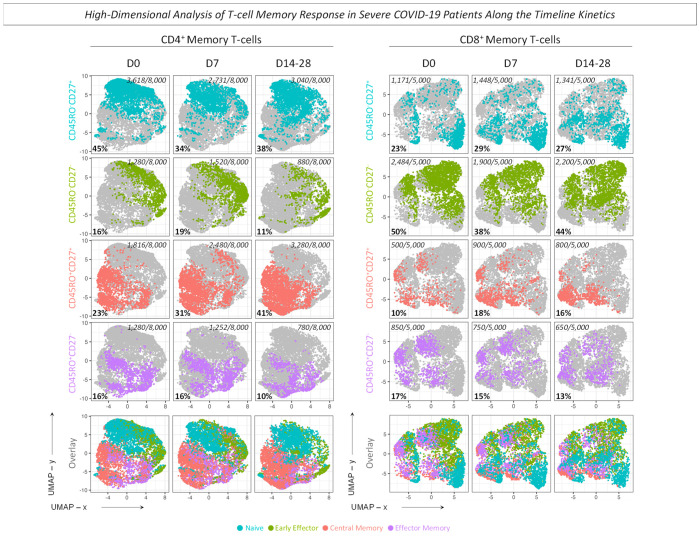
High-dimensional analysis of T-cell memory response in severe COVID-19 patients along the timeline kinetics. Data dimensionality reduction was performed by the UMAP algorithm for selected T-cell memory subsets. The cell frequency values were extracted from FlowJo software, and the graphical representation shows the timeline kinetics (D0, D7, D14–28) of 8.000 events for T CD4^+^ and 5.000 events for T CD8^+^ memory populations. The overall frequency of each cell memory subset [Naive/CD45RO^−^CD27^+^ (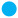), early Effector/CD45RO^−^CD27^−^ (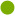), Central Memory/CD45RO^+^CD27^+^ (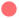), Effector Memory/CD45RO^+^CD27^−^ (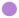)] and the combined clusters off all memory subsets are illustrated in the dotted clusters, while the numbers of frequencies are represented in the UMAP x vs. UMAP y axes.

Complementary analysis was performed using UMAP to visualize the high-dimensional data of selected T-cell phenotypes, as described in Material and Methods (Figure 10 and Figure 11). The results illustrated by the clusters presented an increase for CD4^+^ T-cells at the time point D7 for CD62L^−^, CD27^+^, PD-1^+^, and CD45RO^+^. For CD8^+^ T-cells, data showed the same increase at D7 for PD-1^+^ and CD27^+^. On the other hand, a progressive increase along the timeline kinetics was observed for CD62L^−^ and CD45RO^+^ cell subsets (Figure 10). Additionally, the profile for CD4^+^ T-cell memory subsets showed an increase for the early Effector and a decrease for Naive phenotypes at D7. Conversely, a progressive increase in Central Memory and a progressive reduction in Effector Memory phenotypes were observed. Moreover, The CD8^+^ T-cell memory subsets presented, at D7, an increase for Naive and Central Memory and a decrease for early Effector phenotypes, and the Effector Memory showed a progressive decline along the timeline kinetics (Figure 11). Interestingly, the profile observed in UMAP corroborates with the results illustrated in Figure 5 for the selected cell subsets.

### 3.6. Performance Indices and Binary Logistic Regression Analysis of Cell Phenotypes 

Additional analysis was carried out to evaluate the performance indices (Se, Sp, and AUC) for cell phenotypes along the timeline kinetics for the COVID-19 group and its subgroups according to disease outcome (Discharge and Death), and data are presented in Tables S2 and S3. The results demonstrated that, at D0, nine cell subsets presented robust global accuracy (AUC ≥ 0.8) to classify COVID-19 vs. HCs, with outstanding performance (AUC = 0.9) observed for CD8^+^CD69^+^, CD4^+^CD38^+^, CD3^+^CD38^+^ and CD8^+^CD27^+^. At D7, 13 cell subsets presented remarkable global accuracy (AUC ≥ 0.8), with perfect performance (AUC = 1.0) registered for CD8^+^CD69^+^, CD4^+^CD38^+^ and AUC = 0.9 for CD8^+^CD107a^+^ and CD4^+^T-bet^+^. Later at D14–28, 16 cell subsets presented elevated global accuracy (AUC ≥ 0.8) to classify COVID-19 vs. HCs, with AUC = 1.0 registered for CD4^+^CD38^+^ and CD8^+^CD69^+^ and AUC = 0.9 observed for CD8^+^CD27^+^, CD3^+^CD107a^+^, CD4^+^T-bet^+^, CD8^+^CD107a^+^ and CD3^+^CD38^+^. Overall, our findings demonstrated that, regardless of the time points along the timeline kinetics, CD4^+^CD38^+^ and CD8^+^CD69^+^ presented outstanding accuracy (AUC ≥ 0.9) in classifying COVID-19 patients vs. HCs. Moreover, CD8^+^CD107a^+^ and CD4^+^T-bet^+^ appeared as relevant cell subsets to classify COVID-19 patients vs. HCs from D7 throughout D14–28 (Table S2). 

Moreover, a small set of cell phenotypes could classify COVID-19 patients according to the disease outcome. At D0, CD3^+^ displayed moderate accuracy (AUC = 0.8). Later, at D7, four cell subsets presented elevated global accuracy (AUC ≥ 0.8) to classify disease outcome, with outstanding performance (AUC ≥ 0.9) reported for CD4^+^TIM-3^+^ and CD3^+^TIM-3^+^ (Table S3).

Aiming to identify risk factors for disease outcome, the relationship between cell phenotype profiles and discharge or death outcomes was assessed along the timeline kinetics by binary logistic regression analysis, and the results are shown in Table 1. Data analysis demonstrated that 11 cell subsets exhibited significant OR values for disease outcome at D0, with the patients’ high percentages of CD4^+^CD107a^+^, CD4^+^T-bet^+^, and CD8^+^T-bet^+^ presenting a 27%, 23%, and 17% increase in the odds of death outcome, respectively. Conversely, at D7, three cell subsets showed significant OR values for disease outcome, with patients with a high percentage of CD4^+^CD45RO^+^CD27^+^ presenting a 6% decrease in the odds of death outcome. Overall, regardless of the significance of OR values obtained for these cell phenotypes, the predictive strength of the association was considered low to identify distinct medical outcomes (Table 1).

## 4. Discussion

The global pandemic of coronavirus disease-2019 (COVID-19), caused by the SARS-CoV-2 virus, brought out the importance of a well-balanced and effective immune response in the host, given the complexity of the response triggered by a life-threatening disease that led to a significant number of fatalities worldwide. In this context, several studies have demonstrated that immune dysregulation can significantly impact disease outcome, leading to severe and potentially fatal conditions [13,15,25].

The SARS-CoV-2 infection has been associated with immune dysfunction during disease pathogenesis and clinical manifestations. In this line, T- and B-cell activation and exhaustion may lead to an impaired immune response, resulting in the expression of stimulatory and inhibitory molecules by T- and B-cells [26]. The overexpression of these cell markers in COVID-19 patients contributes to the pathogenesis and clinical manifestations of the disease and suggests a dysregulated immune response, followed by the inability to support a successful antiviral response. For this reason, understanding the cellular phenotypes of SARS-CoV-2 infection is crucial in regard to developing effective therapeutic strategies to mitigate its effects [9,27].

Despite the end of the pandemic, COVID-19 cases are still being identified worldwide, and, therefore, reliable, easily assessable laboratory markers are valuable as indicators of the course and outcome of COVID-19. In this sense, aiming to provide supportive information about the early and late changes in the cell phenotypic profile of COVID-19 patients, we have assessed the cell composition along the timeline kinetics of phenotypic features of T- and B-cell subsets in severe COVID-19 patients, categorized according to disease outcome.

Our results demonstrated an overall reduction in CD3^+^ T-cells, known as lymphopenia, which is commonly observed in COVID-19 patients and is associated with disease severity, clinical outcomes, and treatment efficacy [14,28,29,30]. Moreover, our results demonstrated that this lymphopenia is followed by increased cell activation and exhaustion markers from COVID-19 patients during the infection, characterizing the dysregulated immune response against SARS-CoV-2. In agreement with our findings, which showed higher frequencies of CD69^+^, CD223^+^, and CD107a^+^ in T-cells, previous studies have demonstrated increased expression of markers associated with T-cell activation (CD69^+^ and CD38^+^) and exhaustion (PD-1^+^ and TIM-3^+^) that can persist beyond the acute phase of COVID-19 infection [9,31,32]. Additionally, other studies showed that COVID-19 patients displayed a strong upregulation of activation and exhaustion markers in T-cells, which is linked with poor clinical outcomes [7,33]. Of note, a higher expression of the CD107a^+^ marker in the lung tissues of COVID-19-infected patients indicates increased frequencies of CD8^+^ T-cell activation [34]. Moreover, severe COVID-19 patients have higher frequencies of CD4^+^ T-cells lacking CD62L expression than non-severe patients, suggesting potential homing to lymph nodes or infected tissues [35]. In addition, the analysis of the B-cell phenotypic profile demonstrated increased frequencies of CD19^+^ cells and CD27^+^ subset. However, decreased frequencies of CD62L^−^ were found in COVID-19 patients. In agreement with these findings, studies have pointed out that CD19^+^CD62L^−^ decreased in disease severity and CD27^+^ remained increased [36,37]. Also, other studies showed an increase in the overall frequencies of B-cells in severe COVID-19 cases compared to mild cases [20,38]. Together, these findings suggest that T- and B-cell dysfunction in response to SARS-CoV-2 may have relevant clinical implications associated with the severity of the disease. It is still an open question whether antibody response in the early weeks of SARS-CoV-2 infection plays a role in controlling disease severity [26]. It has been suggested that the relative targeting of the antibody response to distinct antigens might be associated with different disease severity. However, previous reports have demonstrated that the frequencies of antibody response to SARS-CoV-2 may not be directly associated with the disease severity. In this sense, it is possible that an effective cellular immune response, besides the specific antibody response, is relevant in regard to driving the disease outcome [39]. Our current understanding suggests a relative contribution of T- and B-cell immunity to SARS-CoV-2 as a potential correlate of protection that may have implications for developing effective therapeutic interventions.

The analysis of memory T-cell profiles in COVID-19 patients has demonstrated that CD4^+^ and CD8^+^ T-cells are differentiated into Effector subsets during acute viral infections [19,40]. Lower percentages of early Effector T-cells and Effector Memory T-cells were found in CD8^+^ T-cells of severe patients compared with those of mild patients. Also, the frequency of Naive T-cells in CD8^+^, as well as the frequency of Central Memory T-cells in CD4^+^, were decreased in severe patients [19,40]. Other studies support an overall increase in early Effector and Effector Memory for CD4^+^ and CD8^+^ T-cell subsets in COVID-19 patients [41,42,43,44,45]. In consonance, our results demonstrated higher frequencies of early Effector and Effector Memory T-cells and increased Naive cells in the CD4^+^ T-cells subset. In contrast, CD8^+^ T-cells showed a decreased percentage of early Effector and Central memory subsets. 

In the present study, the timeline kinetics signatures of cell phenotypes were constructed to address the cellular profile during COVID-19. The results demonstrated that, at D7, a higher number of alterations are observed amongst all cell phenotypes, including T- and B-cells with activation, exhaustion, and memory profiles. These data suggest that an evident immune dysregulation occurs at the D7 time point. The analysis of integrative networks between T- and B-cells showed an increase in correlations along the timeline kinetics, with a higher number of correlations at the D14–28 time point. These findings suggest that, following the loss of correlations observed at D0, there is a recovery in cell connectivity at D14–28, indicating a remodeling of the immune response backward to the profile observed in health controls.

The clinical outcomes during SARS-CoV-2 infection may vary depending on disease severity, pre-existing health conditions, and the functional capacity of the immune cellular response, such as lymphopenia [25,29]. Furthermore, the application of a computational approach integrating deep immune profiling with information related to disease severity trajectory and other clinical information revealed that a distinct immunological profile is linked to distinct clinical outcomes [27]. Also, it was observed that T-cell responses may present a hyperactivated or altered differentiation state in SARS-CoV-2 infection [19]. In the present study, two kinetic profiles of T- and B-cell phenotypes were observed to be associated with distinct outcomes of COVID-19. An unimodal peak around D7 was observed for T-cells in both clinical outcomes, whereas an opposite pattern of B-cells was identified in patients progressing to death.

Moreover, our data shows that patients evolving to death generally presented higher frequency CD69^+^, CD223^+^, CD107a^+^, T-bet^+^, and PD-1^+^ subsets. Meanwhile, some studies have demonstrated an association between increased expression of effector molecules by CD8^+^ T-cells in acute COVID-19 and improved clinical outcomes [17,46]. Another relevant point was that high frequencies of T-cell activation, however, negatively impact clinical outcomes. It is well known that polyfunctionality of T-cells is associated with moderate disease, but exacerbated frequencies of T-cell activation appear to be harmful [17]. In some cases, the expression of cell markers associated with potential exhaustion, such as PD-1 and TIM-3, has been associated with disease progression. However, this may not necessarily reflect functional impairment but ongoing exacerbated activation [9,27,47]. Although the integrative network analysis demonstrated a progressive increase in correlations during the timeline kinetics in patients progressing to discharge or death, a higher number of correlations were observed in patients evolving to discharge, suggesting that there is an initial loss of correlations at D0 followed by a recovery along the course of infection towards D14–28.

The mechanisms underlying the hyperactivation and exhaustion in COVID-19 have not been thoroughly characterized, and differences in the timing of systemic and organ-specific immune response may represent a key to understanding the physiopathology of the disease outcome. Distinguishing the mechanisms of immune response associated with protection and pathology induced by SARS-CoV-2 infection are essential guidelines for clinical treatment and immune prevention. Previous studies revealed that SARS-CoV-2 infection triggered a hyperinflammatory state and caused immune exhaustion by directly infecting the host’s immune cells. The disequilibrium between activation/exhaustion of T- and B-cells can result in uncontrolled infection, clinically manifested as critical illness or even death. Apart from lymphopenia, part of the unbalanced adaptive immune response in patients with severe COVID-19 evolving to death was accompanied by exhaustion phenotypes of T-cells. Inhibitory receptor molecules, such as CD223, highly expressed in CD4^+^ T-cells, and PD-1, expressed by CD4^+^ and CD8^+^ T-cells in peripheral blood cells from patients progressing to death, indicated that SARS-CoV-2 triggers immune escape by inducing T-cell exhaustion during severe COVID-19 [48].

It is important to mention that the present study has some limitations. The small number of patients enrolled re-enforces the need for future investigations to validate our findings. Remarkably, the study was carried out during the circulation of the B.1.1.28 and B.1.1.33 SARS-CoV-2 strains in Brazil; therefore, additional studies, including studies involving other variants, may lead to distinct immunological profiles. Loss to follow-up impacted the prospective longitudinal analysis. The observational design with multiple comparisons without corrections by comorbidities for confounding variables also constituted a limitation. The impact of comorbidities on cellular phenotypes and soluble mediators was not performed in this present investigation. Another limitation that should be related is the fact that COVID-19-discharged patients or those who progressed to death during the timeline kinetics reduced the total number of the study population, characterizing a transversal study. Additional analyses are still required to assess broader information, such as in vitro cell culture using a SARS-CoV-2 inactivated viral antigen to evaluate the phenotype profile after cell stimulation.

In conclusion, in aiming to provide a better understanding of COVID-19 outcome phenotypes, our study has characterized the dynamics of the changes in T- and B-cell subsets during COVID-19 and identified T-cell phenotypes able to predict the risk of discharge or death outcome with the perspective of use in clinical applications. Overall, our findings summarized a set of phenotypes feasible to be considered as potential predictors of COVID-19 outcome, including CD4^+^CD107a^+^, CD4^+^T-bet^+^, CD8^+^CD69^+^, CD8^+^T-bet^+^ and CD4^+^CD45RO^+^CD27^+^. Prospective future research should focus on further investigations to better understand the mechanisms of T- and B-cell disfunction associated with immune injury or protection triggered by SARS-CoV-2 infection to support novel scientific strategies for developing immunotherapies and new vaccines.

## Figures and Tables

**Table 1:** Odds ratio for disease outcome of COVID-19 patients based on the cell phenotype profiles along the timeline kinetics according to disease outcome.

	Disease Outcome—Death vs. Discharge		
Timeline	Cell Subset	Odds Ratio (CI 95%)	*p* Value *
D0	CD3^+^	0.94 (0.91–0.98)	0.000
	CD4^+^	0.94 (0.90–0.98)	0.002
	CD4+CD107a^+^	1.27 (1.00–1.61)	0.031
	CD4^+^T-bet^+^	1.23 (1.03–1.48)	0.009
	CD4^+^CD45RO^+^CD27^+^	0.96 (0.93–0.99)	0.017
	CD4^+^CD45RO^−^CD27^+^	0.97 (0.95–0.99)	0.005
	CD4^+^CD45RO^−^CD27^−^	1.04 (1.01–1.07)	0.001
	CD8^+^CD69^+^	1.11 (1.01–1.23)	0.011
	CD8^+^T-bet^+^	1.17 (1.04–1.31)	0.001
	CD8^+^CD27^+^	0.98 (0.95–0.99)	0.037
	CD8^+^CD45RO^−^CD27^+^	0.97 (0.95–0.99)	0.011
D7	CD4^+^CD45RO^+^	0.94 (0.89–0.99)	0.017
	CD4^+^CD45RO^+^CD27^+^	0.94 (0.89–0.99)	0.003
	CD8^+^	0.89 (0.81–0.99)	0.021
D14–28	CD4^+^	0.86 (0.76–0.98)	0.006

Odds ratio was calculated by binary logistic regression. * Significance was considered at *p* < 0.05.

## Data Availability

The original contributions presented in the study are included in the article/Supplementary Materials. Further inquiries can be directed to the corresponding author.

## Supplementary Materials

The following supporting information can be downloaded at: https://www.mdpi.com/article/10.3390/microorganisms12112272/s1, Figure S1: Representative Gating Strategies for Phenotypic Analysis of T and B-cells. Ex vivo immunophenotypic staining of peripheral blood leukocytes was carried out by flow cytometry as described in Material and Methods. Representative pseudocolor density plots illustrate the gating strategies employed for the analysis of T and B-cells. The events were first gated by Time vs. Side Scatter Area (SSC-A) to monitor the acquisition quality based on the continuous laser scatter profile. Following, single cells were selected according to Forward Scatter Area (FSC-A) vs. Forward Scatter Height (FSC-H) distribution. After that, lymphocytes were gated based on their size (FSC-A) and granularity (SSC-A) properties. Phenotypic analyses of T and B-cells were further assessed based on fluorescent-labeling with monoclonal antibodies. For B-cell analysis, CD19^+^ events were selected for further assessment of complementary immunophenotypic features (e.g., PD-1^+^, CD62L^−^). For T-cell analysis, CD3^+^ events were gated for subsequent assessment of complementary immunophenotypic features (e.g., PD-1^+^, CD62L^−^). Analysis of T-cell subsets was carried out by first gating CD4^+^ and CD8^+^ events prior to the assessment of complementary immunophenotypic features (e.g., PD-1^+^, CD62L^−^) or memory phenotypes (e.g., CD45RO^+^ and CD27^+^); Figure S2: Phenotypic Profile of CD4^+^ and CD8^+^ T-cell Memory Subsets in Severe COVID-19 Patients at Baseline (D0). Phenotypic profile of CD4^+^ and CD8^+^ memory T-cell subsets (naïve/CD45RO^−^CD27^+^, early effector/CD45RO^−^CD27^−^, central memory/CD45RO^+^CD27^+^ and effector memory/CD45RO^+^CD27^−^) was assessed in peripheral blood samples collected from COVID-19 patients at baseline (, n = 87) and healthy controls (, HC, n = 13). Immunophenotypic staining was carried out as described in Material and Methods. Data are shown as scattering distribution of individual values over bar charts representing the median percentage (%) of gated cells. Comparative analyses between COVID-19 vs. HC were performed by Mann-Whitney test and significant differences are indicated by connecting lines and asterisks for *p* < 0.01 (**) and *p* < 0.05 (*). Colored backgrounds underscore decreased (), increased () or unaltered () percentages of cell subsets in COVID-19 as compared to HC; Figure S3: Phenotypic Profile of CD4^+^ and CD8^+^ T-cell Memory Subsets in Severe COVID-19 Patients at Baseline (D0) According to Disease Outcome. Phenotypic profile of CD4^+^ and CD8^+^ memory T-cell subsets (naïve/CD45RO^−^CD27^+^, early effector/CD45RO^−^CD27^−^, central memory/CD45RO^+^CD27^+^ and effector memory/CD45RO^+^CD27^−^) was assessed in peripheral blood samples collected from COVID-19 patients at baseline (n = 71) further categorized according to disease outcome to Discharge (, n = 38) or Death (, n = 33) and compared with the reference range (25th–75th interquartile) of healthy controls (HC, n = 13, dashed lines). Immunophenotypic staining was carried out as described in Section 2. Data are shown bar charts representing the median percentage (%, 95% IC) of gated cells. Comparative analysis amongst COVID-19 subgroups and HC were performed by Kruskal-Wallis test followed by Dunn’s post-test for multiple comparisons. Significant differences are underscored by # for comparisons with HC and by connecting lines and asterisks for comparative analysis between COVID-19 subgroups at *p* < 0.001 (***), *p* < 0.01 (**) and *p* < 0.05 (*). Color backgrounds underscore decreased (), increased () or unaltered () percentages of cell subsets in COVID-19 subgroups as compared to HC. Color frames highlight decreased () or increased () percentages of cell subsets in COVID-19 patients progressing to Death as compared to those evolving to Discharge; Figure S4: Kinetics Timeline Signatures and Cell Phenotype Rhythm in Severe COVID-19 Patients According to Days of Symptoms at Admission. The timeline kinetics profile of ex vivo phenotypic features of T-cells, B-cells and T-cell subsets was assessed in peripheral blood samples collected from COVID-19 patients at distinct time points according to days of symptoms at admission, referred to as: “3–10 Days” and “11–24 Days” after symptoms onset. Timeline kinetics profile was assessed at baseline (D0, n = 17 and n = 18, respectively), seven days (D7, n = 16 and n = 15, respectively) and 14–28 days (D14-28, n = 9 and n = 18, respectively) after inclusion in the study and compared with healthy controls (HC, n = 13). Data analyses were carried out by converting the median percentage of gated cells into Z-score as described in Material and Methods. Heatmaps were assembled to underscore the cell subsets with Z-score below or above 0.5 according to the color key provided in the figure. The number of cell subsets with Z-score above 0.5 was calculated and data were shown in line charts to illustrate the cell phenotype rhythm along the days after inclusion. Colored backgrounds underscore decreased () or increased () numbers of cell subsets in COVID-19 as compared to the reference values observed in HC (continuous line); Figure S5: Common Profile of Cell Phenotype Subsets Along the Kinetics Timeline in Severe COVID-19 Patients According to Disease Outcome. The timeline kinetics signature profile of ex vivo phenotypic features of T-cells, B-cells and T-cell subsets were used to construct volcano plots. Data analyses were carried out by Fisher’s Exact test and p values were expressed in negative log_10_ (−log_10_p). Delta was calculated by the comparisons between HC vs outcome subgroups Discharge and Death (ΔHC). Cell phenotypes above the cut-off (1.3 −log_10_p) were highlighted by green () and red () dots representing decrease and increase of ΔHC values, respectively. Grey dots () were used to identify cell phenotypes below the cut-off. The cell phenotypes that are exclusive for each time point are in bold and underlined text. The cell phenotypes that are common in more than one time point are presented in grey color, and the cell phenotypes that are common between all the time points are listed below the graphs in green (decrease) and red (increase); Table S1: Hematological profile of COVID-19 patients at baseline (D0) according to disease outcome; Table S2: Overall performance of cellular phenotypes from COVID-19 patients along the timeline kinetics; Table S3: Overall performance of cellular phenotypes from COVID-19 patients along the timeline kinetics according to disease outcome.

## References

[1] Vardhana S.A., Wolchok J.D. (2020). The Many Faces of the Anti-COVID Immune Response. J. Exp. Med..

[2] Wang Y., Wang Y., Chen Y., Qin Q. (2020). Unique Epidemiological and Clinical Features of the Emerging 2019 Novel Coronavirus Pneumonia (COVID-19) Implicate Special Control Measures. J. Med. Virol..

[3] Bornstein S.R., Dalan R., Hopkins D., Mingrone G., Boehm B.O. (2020). Endocrine and Metabolic Link to Coronavirus Infection. Nat. Rev. Endocrinol..

[4] Gharebakhshi F., Abbasian S., Shariati Sough M., Zaremoghadam E., Zandiyeh F., Abdolmohammadi G., Zarei A., Tavassoli Z., Kalirad A., Belali Kharaji M. (2023). Pulmonary Radiologic Findings Based on Warrick Score as a Predictor of COVID-19 Patients’ Outcomes. Immunopathol. Persa.

[5] Kragstrup T.W., Singh H.S., Grundberg I., Nielsen A.L.-L., Rivellese F., Mehta A., Goldberg M.B., Filbin M.R., Qvist P., Bibby B.M. (2021). Plasma ACE2 Predicts Outcome of COVID-19 in Hospitalized Patients. PLoS ONE.

[6] Jarjour N.N., Masopust D., Jameson S.C. (2021). T Cell Memory: Understanding COVID-19. Immunity.

[7] Herrmann M., Schulte S., Wildner N.H., Wittner M., Brehm T.T., Ramharter M., Woost R., Lohse A.W., Jacobs T., Schulze zur Wiesch J. (2020). Analysis of Co-Inhibitory Receptor Expression in COVID-19 Infection Compared to Acute Plasmodium Falciparum Malaria: LAG-3 and TIM-3 Correlate with T Cell Activation and Course of Disease. Front. Immunol..

[8] Hanna S.J., Codd A.S., Gea-Mallorqui E., Scourfield D.O., Richter F.C., Ladell K., Borsa M., Compeer E.B., Moon O.R., Galloway S.A.E. (2021). T Cell Phenotypes in COVID-19—A Living Review. Oxf. Open Immunol..

[9] Diao B., Wang C., Tan Y., Chen X., Liu Y., Ning L., Chen L., Li M., Liu Y., Wang G. (2020). Reduction and Functional Exhaustion of T Cells in Patients with Coronavirus Disease 2019 (COVID-19). Front Immunol..

[10] Tarique M., Suhail M., Naz H., Muhammad N., Tabrez S., Zughaibi T.A., Abuzenadah A.M., Hashem A.M., Shankar H., Saini C. (2022). Where Do T Cell Subsets Stand in SARS-CoV-2 Infection: An Update. Front. Cell Infect. Microbiol..

[11] Modabber Z., Shahbazi M., Akbari R., Bagherzadeh M., Firouzjahi A., Mohammadnia-Afrouzi M. (2021). TIM-3 as a Potential Exhaustion Marker in CD4^+^ T Cells of COVID-19 Patients. Immun. Inflamm. Dis..

[12] Bobcakova A., Barnova M., Vysehradsky R., Petriskova J., Kocan I., Diamant Z., Jesenak M. (2022). Activated CD8^+^CD38^+^ Cells Are Associated with Worse Clinical Outcome in Hospitalized COVID-19 Patients. Front. Immunol..

[13] Tarbiah N.I., Alkhattabi N.A., Alsahafi A.J., Aljahdali H.S., Joharjy H.M., Al-Zahrani M.H., Sabban A.M., Alghamdi R.A., Balgoon M.J., Khalifa R.A. (2023). T Cells Immunophenotyping and CD38 Overexpression as Hallmarks of the Severity of COVID-19 and Predictors of Patients’ Outcomes. J. Clin. Med..

[14] Wang L., Chen J., Zhao J., Li F., Lu S., Liu P., Liu X., Huang Q., Wang H., Xu Q. (2021). The Predictive Role of Lymphocyte Subsets and Laboratory Measurements in COVID-19 Disease: A Retrospective Study. Ther. Adv. Respir. Dis..

[15] Niedźwiedzka-Rystwej P., Majchrzak A., Aksak-Wąs B., Serwin K., Czajkowski Z., Grywalska E., Korona-Głowniak I., Roliński J., Parczewski M. (2022). Programmed Cell Death-1/Programmed Cell Death-1 Ligand as Prognostic Markers of Corona virus Disease 2019 Severity. Cells.

[16] Li S., Wang Y., Feng L., People’s Hospital N., Jiang Z., Chen Y., Dai Z., Liu S., Zhu S., Fei Z. (2020). Chemokine Receptor Inhibitor VMIP-II Promoting Lymphocytes in COVID-19 Patients and Its Related Mechanism In Vitro. Res. Sq..

[17] Su Y., Chen D., Yuan D., Lausted C., Choi J., Dai C.L., Voillet V., Duvvuri V.R., Scherler K., Troisch P. (2020). Multi-Omics Resolves a Sharp Disease-State Shift between Mild and Moderate COVID-19. Cell.

[18] Chen Z., John Wherry E. (2020). T Cell Responses in Patients with COVID-19. Nat. Rev. Immunol..

[19] Kalpakci Y., Hacibekiroglu T., Trak G., Karacaer C., Demirci T., Kocayigit H., Sunu C., Varim C., Falay M. (2020). Comparative Evaluation of Memory T Cells in COVID-19 Patients and the Predictive Role of CD4^+^CD8^+^ Double Positive T Lymphocytes as a New Marker. Rev. Assoc. Med. Bras..

[20] Sosa-Hernández V.A., Torres-Ruíz J., Cervantes-Díaz R., Romero-Ramírez S., Páez-Franco J.C., Meza-Sánchez D.E., Juárez-Vega G., Pérez-Fragoso A., Ortiz-Navarrete V., Ponce-de-León A. (2020). B Cell Subsets as Severity-Associated Signatures in COVID-19 Patients. Front. Immunol..

[21] Sallusto F., Geginat J., Lanzavecchia A. (2004). Central memory and effector memory T cell subsets: Function, generation, and maintenance. Annu. Rev. Immunol..

[22] Marín N.D., París S.C., Rojas M., García L.F. (2012). Reduced frequency of memory T cells and increased Th17 responses in patients with active tuberculosis. Clin. Vaccine Immunol..

[23] Moura R.A., Quaresma C., Vieira A.R., Gonçalves M.J., Polido-Pereira J., Romão V.C., Martins N., Canhão H., Fonseca J.E. (2017). B-cell phenotype and IgD-CD27- memory B cells are affected by TNF-inhibitors and tocilizumab treatment in rheumatoid arthritis. PLoS ONE.

[24] Castleman M.J., Santos A.L., Lesteberg K.E., Maloney J.P., Janssen W.J., Mould K.J., Beckham J.D., Pelanda R., Torres R.M. (2023). Activation and pro-inflammatory cytokine production by unswitched memory B cells during SARS-CoV-2 infection. Front. Immunol..

[25] Gonçalves J.J., da Mata C.P.S.M., Lourenço A.A., Ribeiro Á.L., Ferreira G.M., Fraga-Silva T.F.d.C., de Souza F.M., Almeida V.E.S., Batista I.A., D’Avila-Mesquita C. (2022). Timeline Kinetics of Systemic and Airway Immune Mediator Storm for Comprehensive Analysis of Disease Outcome in Critically Ill COVID-19 Patients. Front. Immunol..

[26] Röltgen K., Powell A.E., Wirz O.F., Stevens B.A., Hogan C.A., Najeeb J., Hunter M., Wang H., Sahoo M.K., Huang C. (2020). Defining the features and duration of antibody responses to SARS-CoV-2 infection associated with disease severity and outcome. Sci. Immunol..

[27] Mathew D., Giles J.R., Baxter A.E., Oldridge D.A., Greenplate A.R., Wu J.E., Alanio C., Kuri-Cervantes L., Pampena M.B., D’Andrea K. (2020). Deep Immune Profiling of COVID-19 Patients Reveals Distinct Immunotypes with Therapeutic Implications. Science.

[28] Delshad M., Tavakolinia N., Pourbagheri-Sigaroodi A., Safaroghli-Azar A., Bagheri N., Bashash D. (2021). The Contributory Role of Lymphocyte Subsets, Pathophysiology of Lymphopenia and Its Implication as Prognostic and Therapeutic Opportunity in COVID-19. Int. Immunopharmacol..

[29] Lee J., Park S.-S., Kim T.Y., Lee D.-G., Kim D.-W. (2021). Lymphopenia as a Biological Predictor of Outcomes in COVID-19 Patients: A Nationwide Cohort Study. Cancers.

[30] Suryawanshi P., Takbhate B., Athavale P., Jali P., Memane N., Mirza S., Karandikar M., Kakrani A.L., Kanitkar S., Gandham N. (2023). Lymphopenia with Altered T Cell Subsets in Hospitalized COVID-19 Patients in Pune, India. Viral Immunol..

[31] Trøseid M., Dahl T.B., Holter J.C., Kildal A.B., Murphy S.L., Yang K., Quiles-Jiménez A., Heggelund L., Müller K.E., Tveita A. (2022). Persistent T-cell Exhaustion in Relation to Prolonged Pulmonary Pathology and Death after Severe COVID-19: Results from Two Norwegian Cohort Studies. J. Intern. Med..

[32] Govender M., Hopkins F.R., Göransson R., Svanberg C., Shankar E.M., Hjorth M., Nilsdotter-Augustinsson Å., Sjöwall J., Nyström S., Larsson M. (2022). T Cell Perturbations Persist for at Least 6 Months Following Hospitalization for COVID-19. Front. Immunol..

[33] Mitsuyama Y., Yamakawa K., Kayano K., Maruyama M., Wada T., Fujimi S. (2021). Prolonged Enhancement of Cytotoxic T Lymphocytes in the Post-Recovery State of Severe COVID-19. J. Intensive Care.

[34] Tan X., Grice L.F., Tran M., Mulay O., Monkman J., Blick T., Vo T., Simões A.C., Almeida F., Da J. (2023). A Robust Platform for Integrative Spatial Multi-Omics Analysis to Map Immune Responses to SARS-CoV-2 Infection in Lung Tissues. Immunology.

[35] Lesteberg K.E., Araya P., Waugh K.A., Chauhan L., Espinosa J.M., Beckham J.D. (2023). Severely Ill and High-Risk COVID-19 Patients Exhibit Increased Peripheral Circulation of CD62L+ and Perforin+ T Cells. Front. Immunol..

[36] Onodera T., Sax N., Sato T., Adachi Y., Kotaki R., Inoue T., Shinnakasu R., Nakagawa T., Fukushi S., Terooatea T. (2023). CD62L Expression Marks SARS-CoV-2 Memory B Cell Subset with Preference for Neutralizing Epitopes. Sci. Adv..

[37] Mansourabadi A.H., Aghamajidi A., Dorfaki M., Keshavarz F., Shafeghat Z., Moazzeni A., Arab F.L., Rajabian A., Roozbehani M., Falak R. (2023). B Lymphocytes in COVID-19: A Tale of Harmony and Discordance. Arch. Virol..

[38] Wang F., Nie J., Wang H., Zhao Q., Xiong Y., Deng L., Song S., Ma Z., Mo P., Zhang Y. (2020). Characteristics of Peripheral Lymphocyte Subset Alteration in COVID-19 Pneumonia. J. Infect. Dis..

[39] Cox R.J., Brokstad K.A. (2020). Not just antibodies: B cells and T cells mediate immunity to COVID-19. Nat. Rev. Immunol..

[40] Zhao B., Zhong M., Yang Q., Hong K., Xia J., Li X., Liu Y., Chen Y.-Q., Yang J., Huang C. (2021). Alterations in Phenotypes and Responses of T Cells Within 6 Months of Recovery from COVID-19: A Cohort Study. Virol. Sin..

[41] Tripathy A., Trimbake D., Suryawanshi P., Tripathy S., Gurav Y., Potdar V., Chaudhary M., Athavale P., Mokashi N., Patsute S. (2022). Peripheral Lymphocyte Subset Alteration in Patients with COVID-19 Having Differential Clinical Manifestations. Indian J. Med. Res..

[42] Yang J., Zhong M., Zhang E., Hong K., Yang Q., Zhou D., Xia J., Chen Y.-Q., Sun M., Zhao B. (2021). Broad Phenotypic Alterations and Potential Dysfunction of Lymphocytes in Individuals Clinically Recovered from COVID-19. J. Mol. Cell Biol..

[43] Kaaijk P., Olivo Pimentel V., Emmelot M.E., Poelen M.C.M., Cevirgel A., Schepp R.M., den Hartog G., Reukers D.F.M., Beckers L., van Beek J. (2022). Children and Adults with Mild COVID-19: Dynamics of the Memory T Cell Response up to 10 Months. Front. Immunol..

[44] Yang J., Zhang E., Zhong M., Yang Q., Hong K., Shu T., Zhou D., Xiang J., Xia J., Zhou X. (2020). Longitudinal Characteristics of T Cell Responses in Asymptomatic SARS-CoV-2 Infection. Virol. Sin..

[45] Yang J., Zhong M., Hong K., Yang Q., Zhang E., Zhou D., Xia J., Chen Y., Sun M., Zhao B. (2021). Characteristics of T-cell Responses in COVID-19 Patients with Prolonged SARS-CoV-2 Positivity—A Cohort Study. Clin. Transl. Immunol..

[46] Notarbartolo S., Ranzani V., Bandera A., Gruarin P., Bevilacqua V., Putignano A.R., Gobbini A., Galeota E., Manara C., Bombaci M. (2021). Integrated Longitudinal Immunophenotypic, Transcriptional, and Repertoire Analyses Delineate Immune Responses in Patients with COVID-19. Sci. Immunol..

[47] Rha M.-S., Shin E.-C. (2021). Activation or Exhaustion of CD8+ T Cells in Patients with COVID-19. Cell Mol. Immunol..

[48] Sun Y., Luo B., Liu Y., Wu Y., Chen Y. (2023). Immune damage mechanisms of COVID-19 and novel strategies in prevention and control of epidemic. Front. Immunol..

