# Supplementary material for: Phenotypic Timeline Kinetics, Integrative Networks, and Performance of T- and B-Cell Subsets Associated with Distinct Clinical Outcome of Severe COVID-19 Patients
Source: Microorganisms. 2024 Nov 9;12(11):2272. doi: 10.3390/microorganisms12112272 (PMC11596994; doi:10.3390/microorganisms12112272)
Supplement: Supplementary file 1 [file microorganisms-12-02272-s001.zip › microorganisms-3058310-supplementary.pdf]

Supplementary Figures

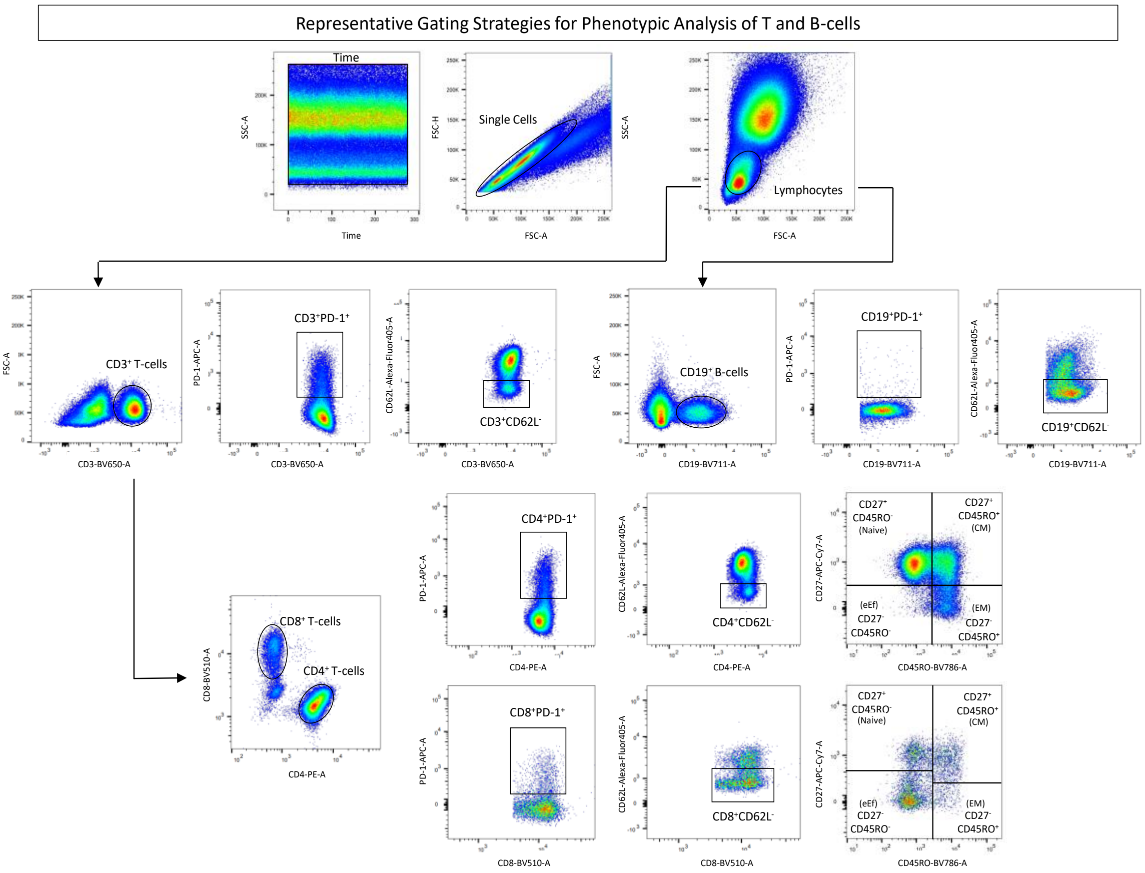

Figure S1. Representative gating strategies for phenotypic analysis of T and B-cells. Ex vivo immunophenotypic staining of peripheral blood leukocytes was carried out by flow cytometry as described in Material and Methods. Representative pseudocolor density plots illustrate the gating strategies employed to analyze T and B-cells. The events were first gated by Time *vs* Side Scatter Area (SSC-A) to monitor the acquisition quality based on the continuous laser scatter profile. Following, single cells were selected according to Forward Scatter Area (FSC-A) *vs* Forward Scatter Height (FSC-H) distribution. After that, lymphocytes were gated based on their size (FSC-A) and granularity (SSC-A) properties. Phenotypic analyses of T and B-cells were further assessed based on fluorescent labeling with monoclonal antibodies. For B-cell analysis, $CD19^{+}$ events were selected for further assessment of complementary immunophenotypic features (e.g. $PD\text{-}1^{+}$, $CD62L^{-}$). For T-cell analysis, $CD3^{+}$ events were gated for the subsequent assessment of complementary immunophenotypic features (e.g. $PD\text{-}1^{+}$, $CD62L^{-}$). Analysis of T-cell subsets was carried out by first gating $CD4^{+}$ and $CD8^{+}$ events before the assessment of complementary immunophenotypic features (e.g. $PD\text{-}1^{+}$, $CD62L^{-}$) or memory phenotypes (e.g. $CD45RO^{+}$ and $CD27^{+}$).

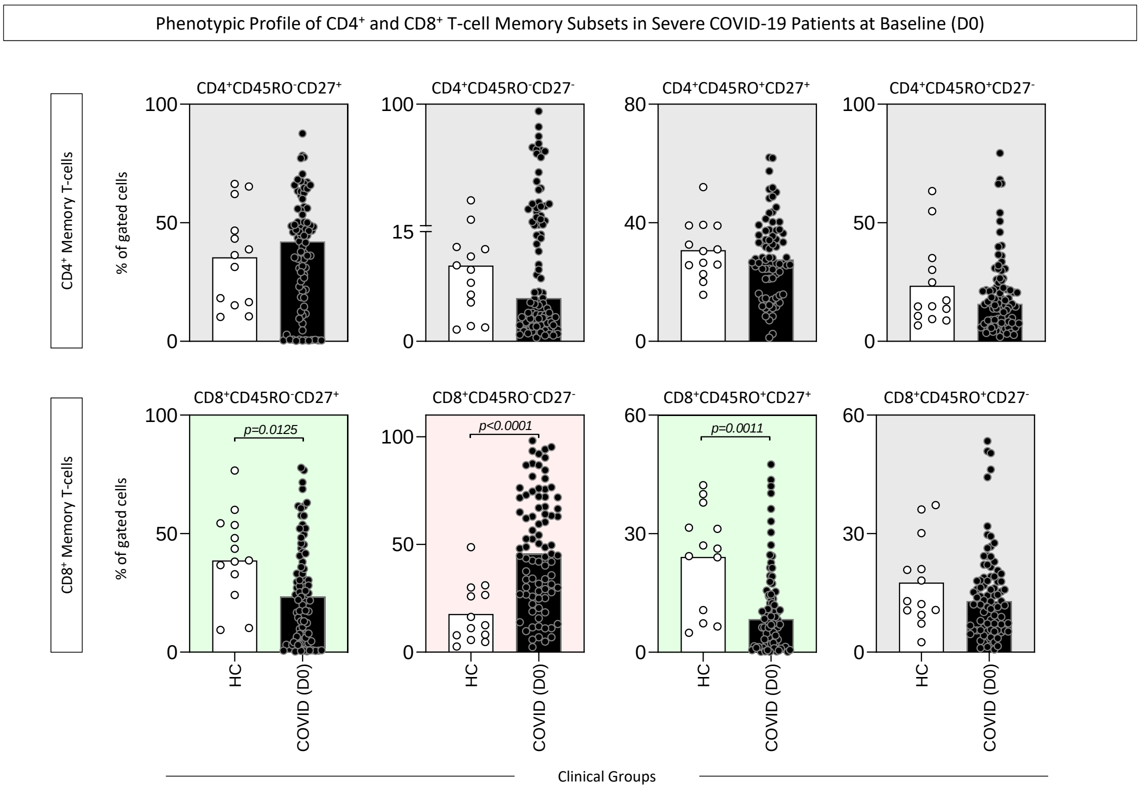

Figure S2. Phenotypic profile of memory CD4⁺ and CD8⁺ T-cell subsets in severe COVID-19 patients at baseline (D0). Phenotypic profile of CD4⁺ and CD8⁺ memory T-cell subsets [Naive/CD45RO⁻CD27⁺, early Effector(eEf)/CD45RO⁻CD27⁻, Central Memory(CM)/CD45RO⁺CD27⁺ and Effector Memory(EM)/CD45RO⁺CD27⁻] was assessed in peripheral blood samples collected from COVID-19 patients at D0 (●, n=87) and healthy controls (○, HC, n=13). Immunophenotypic staining was carried out as described in Material and Methods. Data are shown as scattering distribution of individual values over bar charts representing the median percentage (%) of gated cells. Comparative analyses between COVID-19 *vs.* HC were performed using the Mann-Whitney test, and the p values for significant differences are provided in the figure. Color backgrounds underscore decreased (▭), increased (▭), or unaltered (▭) percentages of cell subsets in COVID-19 as compared to HC.

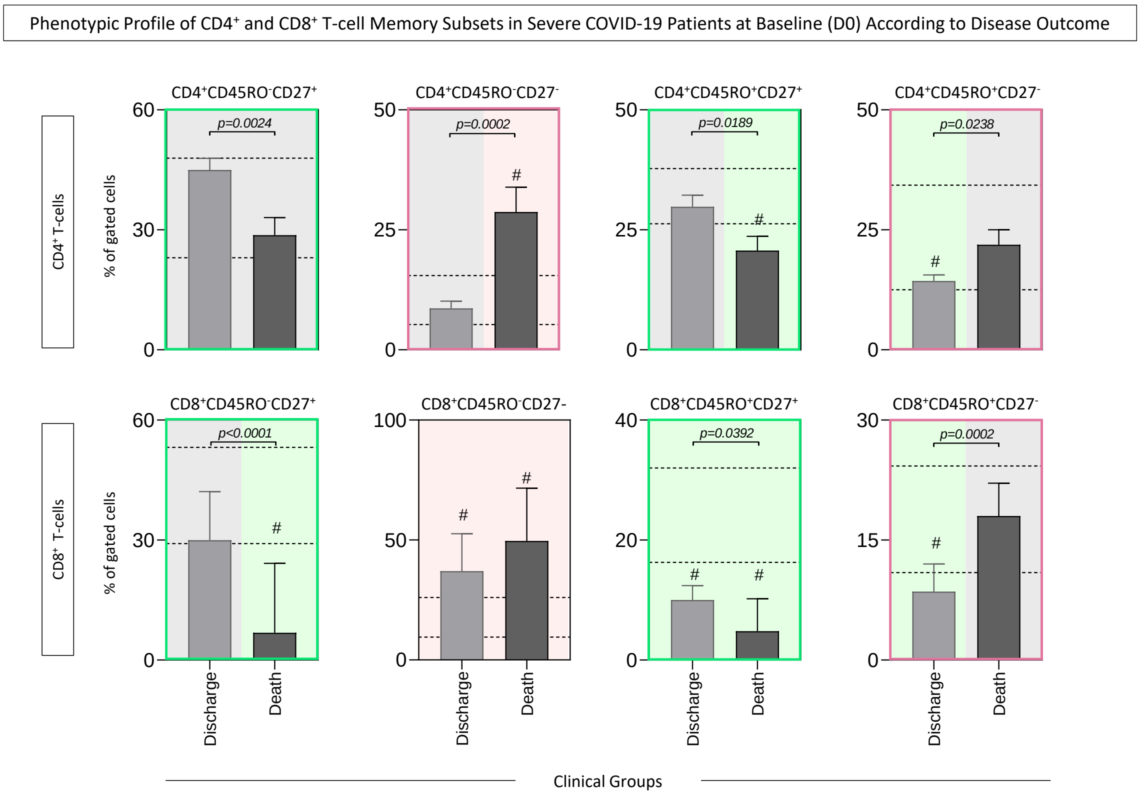

Figure S3. Phenotypic profile of $CD4^+$ and $CD8^+$ memory T-cell subsets in severe COVID-19 patients at baseline (D0) according to disease outcome. Phenotypic profile of $CD4^+$ and $CD8^+$ memory T-cell subsets [Naive/$CD45RO^-CD27^+$, early effector(eEf)/$CD45RO^-CD27^-$, Central Memory(CM)/$CD45RO^+CD27^+$ and Effector Memory(EM)/$CD45RO^+CD27^-$] was assessed in peripheral blood samples collected from COVID-19 patients at D0 (n=71) further categorized according to disease outcome to Discharge (▬, n=38) or Death (▬, n=33) and compared with the reference range (25th-75th interquartile) of healthy controls (HC, n=13, dashed lines). Immunophenotypic staining was carried out as described in Material and Methods. Data are shown as bar charts representing the median percentage (%, 95% IC) of gated cells. Comparative analyses amongst COVID-19 subgroups and HC were performed using the Kruskal-Wallis test, followed by Dunn's post-test for multiple comparisons. Significant differences are underscored by # for comparisons with HC. Significant differences between COVID-19 subgroups were identified by connecting lines, and the p values for significant differences are provided in the figure. Color backgrounds underscore decreased (▭), increased (▭), or unaltered (▭) percentages of cell subsets in COVID-19 subgroups as compared to HC. Color frames highlight decreased (▭) or increased (▭) percentages of cell subsets in COVID-19 patients progressing to Death compared to those evolving to Discharge.

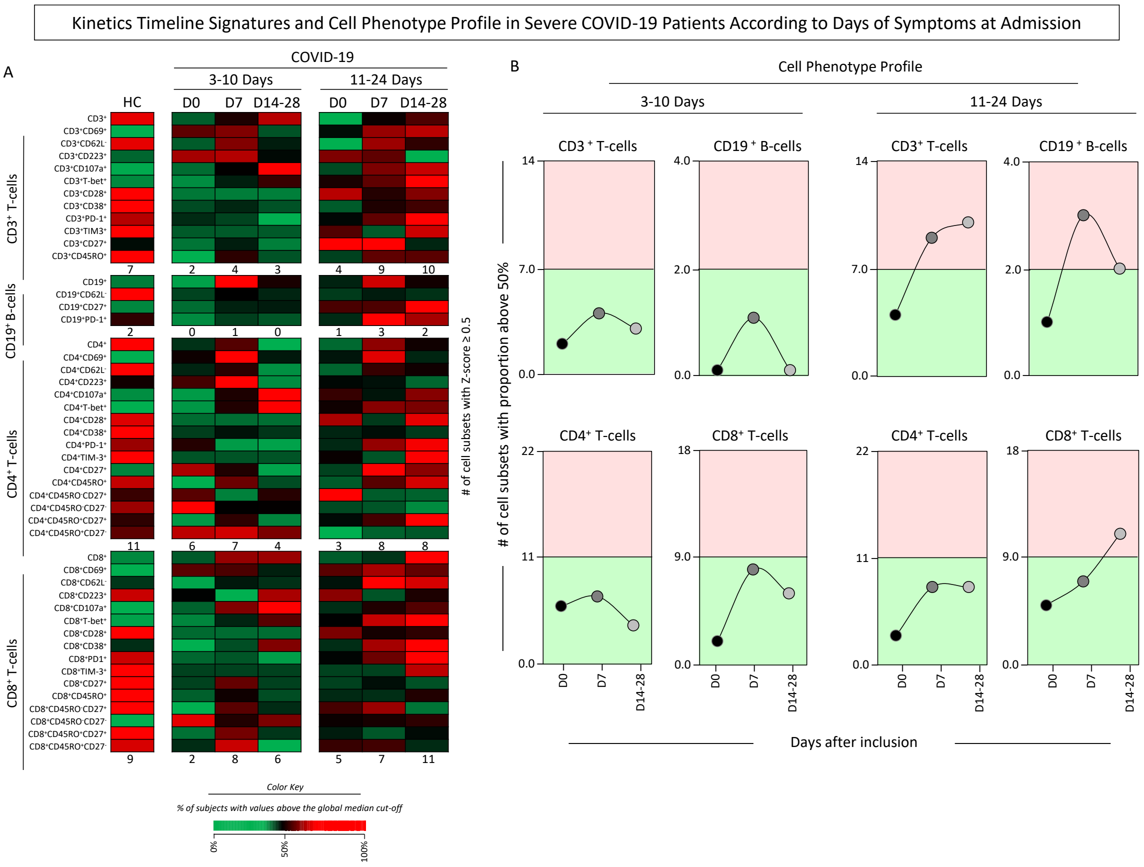

Figure S4. Timeline kinetics of cell phenotype profile in severe COVID-19 patients according to days of symptoms at baseline (D0). The timeline kinetics profile of *ex vivo* phenotypic features of T and B-cell subsets was assessed in peripheral blood samples collected from COVID-19 patients at distinct time points according to days of symptoms onset at admission, referred to as: "3-10 Days" and "11-24 Days". Timeline kinetics profile was assessed at baseline (D0, n=17 and n=18, respectively), seven days (D7, n=16, and n=15, respectively), and 14-28 days (D14-28, n=9, and n=18, respectively) after inclusion in the study and compared with healthy controls (HC, n=13). Data analyses were carried out by converting the median percentage of gated cells into Z-score as described in Material and Methods. (A) Color maps were assembled to underscore the cell subsets with Z-score below or above 0.5 according to the color key provided in the figure. (B) The number of cell subsets with Z-score above 0.5 was calculated and data were shown in line charts to illustrate the cell phenotype profile along the days after inclusion. Color backgrounds underscore decreased (▭) or increased (▭) numbers of cell subsets in COVID-19 as compared to the reference values observed in HC (continuous line).

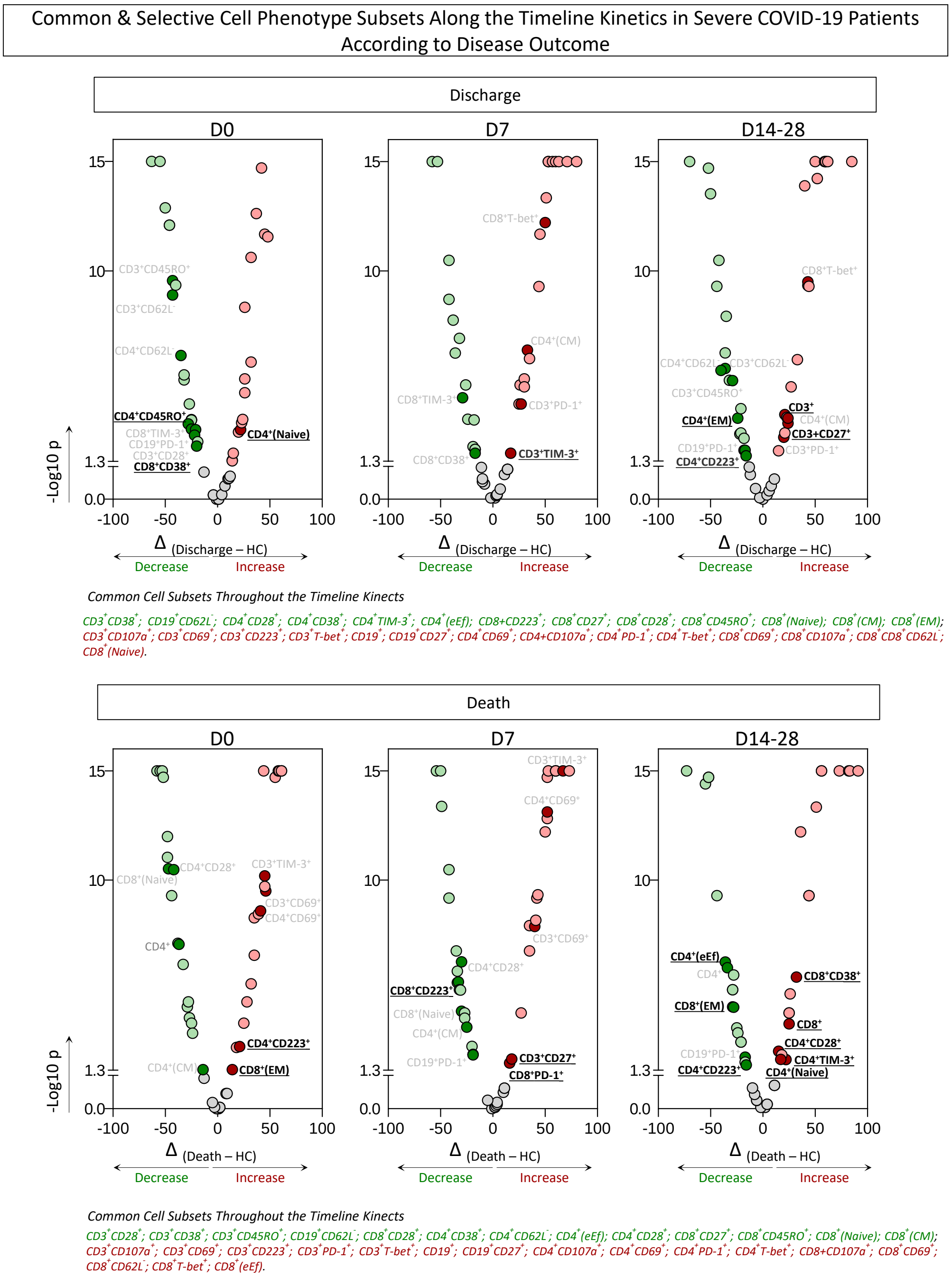

Figure S5. Common & selective cell phenotype subsets along the timeline kinetics in severe COVID-19 patients according to disease outcome. The timeline kinetics signature profile of *ex vivo* phenotypic features of T and B-cell subsets were used to construct volcano plots as scattering distribution of Log10 of fold change of proportion (%) of subjects with results above the global median cut-off in COVID-19 subgroups (Discharge or Death) according to HC [$\Delta_{(Discharge\text{-}HC)}$ and $\Delta_{(Death\text{-}HC)}$] versus significance (-Log10 p values, Fisher's Exact test). Venn diagram analyses were conducted to identify common and selective cell phenotype subsets along the timeline kinetics amongst groups. Cell phenotypes with significant differences at $p<0.05$ (-Log10 p = 1.30103, the gap across the Y axis) are highlighted by green and red dots representing decrease and increase $\Delta_{(Discharge\text{-}HC)}$ and $\Delta_{(Death\text{-}HC)}$ values, respectively. Gray dots are used to identify cell phenotypes without significant differences representing unaltered $\Delta_{(Discharge\text{-}HC)}$ and $\Delta_{(Death\text{-}HC)}$ values. Selective cell phenotypes (dark color dots) are underscored in bold, underline format at each time point. Cell phenotypes shared in more than one time point (dark color dots) are presented in gray format. Common cell phenotypes throughout the timeline kinetics (light color dots) are listed below the graphs and underscored in green (decrease) or red (increase) format.

Supplementary Tables

Table S1 – Hematological profile of COVID-19 patients at baseline (D0) according to disease outcome

Parameters	Groups			
	**HC** (n=13)	**COVID-19** (n=87)	**Discharge** (n=38)	**Death** (n=33)
Hemoglobin [g/dL]	14.5 (13.3-15.4)	11.0# (9.1-12.4)	12.0 (10.4-13.0)	9.7* (7.4-11.2)
RBC [x10⁶/mm³]	5.3 (4.8-5.8)	3.5# (2.9-4.1)	3.9 (3.4-4.3)	3.2* (2.6-3.6)
Hematocrit [%]	48.9 (48.0-51.0)	32.7# (26.7-36.3)	35.0 (31.0-38.6)	28.8* (23.1-34.2)
WBC [x10³/mm³]	5.6 (4.5-7.3)	11.3# (9.0-17.1)	9.8 (6.9-12.6)	18.9* (12.1-23.5)
Granulocytes [x10³/mm³]	0.9 (0.8-1.2)	9.2# (6.7-14.9)	8.1 (5.0-10.9)	15.9* (9.1-21.7)
Lymphocytes [x10³/mm³]	2.1 (1.6-2.6)	1.1# (0.8-1.5)	1.0 (0.8-1.4)	1.2 (0.9-1.7)
Monocytes [x10³/mm³]	0.5 (0.4-0.5)	0.6 (0.3-0.8)	0.4 (0.3-0.6)	0.7* (0.5-1.0)
Gran/Lym [Ratio]	0.4 (0.4-0.5)	9.1# (6.3-16.3)	7.8 (4.2-13.1)	13.9* (7.5-21.6)
Platelets [x10³/mm³]	160 (111-199)	171 (127-224)	181 (140-288)	152* (115-234)

HC = Healthy Controls. COVID-19 = COVID-19 patients at baseline. Discharge and Death = disease outcome. RBC = red blood cells. WBC = white blood cells. Gran = granulocytes. Lym = lymphocytes. Data are reported as median values (95% CI). Significant differences at $p<0.05$ are underscored by # and * as compared to HC and Discharge groups, respectively.

Table S2. Overall performance of cellular phenotypes from COVID-19 patients along the timeline kinetics

				**COVID-19 *vs.* HC**		
**Timeline**	**Cell subset**	**AUC**	**Cut-off**	**Se (95% IC)**	**Sp (95% IC)**	***p value***
**D0**	$CD8^+CD69^+$	0.9 (0.9-1)	> 2.8	89.7 (82-94)	91.7 (64-99)	<0.0001
	$CD4^+CD38^+$	0.9 (0.9-1)	< 22.8	82.8 (73-89)	91.7 (64-99)	<0.0001
	$CD3^+CD38^+$	0.9 (0.8-0.9)	< 20	80.5 (71-87)	91.7 (64-99)	<0.0001
	$CD8^+CD27^+$	0.9 (0.7-0.9)	< 69	93.1 (86-97)	69.2 (42-87)	<0.0001
	$CD8^+CD45RO^-CD27^-$	0.8 (0.7-0.9)	> 31.3	68.2 (58-77)	92.3 (67-99)	0.0001
	$CD8^+CD28^+$	0.8 (0.7-0.9)	< 49.3	71.2 (59-81)	92.3 (67-99)	0.0005
	$CD8^+CD45RO^+$	0.8 (0.6-0.9)	< 30.9	77.7 (68-85)	84.6 (58-97)	0.0011
	$CD8^+CD107a^+$	0.8 (0.7-0.9)	> 0.8	64.7 (54-74)	91.7 (64-99)	0.0018
	$CD8^+CD45RO^+CD27^+$	0.8 (0.6-0.9)	< 23.4	87.1 (78-93)	69.2 (42-87)	0.0015
**D7**	$CD8^+CD69^+$	1.0 (0.9-1.0)	> 3.86	91.9 (79-97)	100.0 (76-100)	<0.0001
	$CD4^+CD38^+$	1.0 (0.9-1.0)	< 22.6	89.5 (76-96))	91.7 (64-99)	<0.0001
	$CD8^+CD107a^+$	0.9 (0.8-1.0)	> 0.9	83.3 (68-92)	91.7 (64-99)	<0.0001
	$CD4^+$T-bet$^+$	0.9 (0.8-1.0)	> 1.9	75.7 (60-87)	100.0 (76-100)	0.0002
	$CD3^+CD38^+$	0.8 (0.7-1.0)	< 19.7	71.1 (55-83)	91.7 (64-99)	0.0003
	$CD4^+CD69^+$	0.8 (0.7-0.9)	> 8.4	57.9 (42-72)	100.0 (75-100)	0.0003
	$CD8^+CD27^+$	0.8 (0.7-0.9)	< 67.9	88.9 (75-95)	69.2 (42-87)	0.0004
	$CD19^+CD27^+$	0.8 (0.7-0.9)	> 10.8	72.2 (56-84)	92.3 (67-99)	0.0005
	$CD3^+CD107a^+$	0.8 (0.7-0.9)	> 1.6	70.3 (54-82)	100.0 (75-100)	0.0007
	$CD3^+$T-bet$^+$	0.8 (0.7-0.9)	> 3.6	67.6 (51-80)	91.7 (64-99)	0.0008
	$CD3^+CD69^+$	0.8 (0.7-0.9)	> 4.5	89.5 (76-96)	66.7 (39-86)	0.001
	$CD8^+CD28^+$	0.8 (0.6-0.9)	< 48.4	66.7 (47-82)	92.3 (67-99)	0.0028
	$CD8^+CD45RO^-CD27^-$	0.8 (0.7-0.9)	> 31.4	58.3 (42-73)	92.3 (67-99)	0.0018
**D14-28**	$CD4^+CD38^+$	1.0 (0.9-1.0)	< 22.7	96.6 (83-1.0)	91.7 (64-99)	<0.0001
	$CD8^+CD69^+$	1.0 (0.9-1.0)	> 3.9	89.3 (73-96)	100.0 (76-100)	<0.0001
	$CD8^+CD27^+$	0.9 (0.8-1.0)	< 54.9	85.2 (67-94)	84.6 (58-97)	<0.0001
	$CD3^+CD107a^+$	0.9 (0.8-1.0)	> 1.6	75.9 (58-88)	100.0 (76-100)	0.0002
	$CD4^+$T-bet$^+$	0.9 (0.7-1.0)	> 2.2	75.9 (58-88)	100.0 (76-100)	0.0002
	$CD8^+CD107a^+$	0.9 (0.7-1.0)	> 0.8	82.1 (64-92)	91.7 (64-99)	0.0003
	$CD3^+CD38^+$	0.9 (0.7-1.0)	< 19.7	65.5 (47-80)	91.7 (64-99)	0.0004
	$CD3^+$T-bet$^+$	0.8 (0.7-1.0)	> 4.0	72.4 (54-85)	100.0 (75-100)	0.0005
	$CD19^+CD27^+$	0.8 (0.7-1.0)	> 11.7	64.0 (44-78)	100.0 (77-100)	0.0006
	$CD8^+CD28^+$	0.8 (0.6-0.9)	< 38.9	61.1 (39-80)	92.3 (67-99)	0.0031
	$CD4^+CD62L^-$	0.8 (0.6-0.9)	< 31.6	80.0 (61-91)	76.9 (50-92)	0.0033
	$CD4^+CD38^+$	1.0 (0.9-1.0)	< 22.7	96.6 (83-1.0)	91.7 (64-99)	<0.0001
	$CD8^+CD69^+$	1.0 (0.9-1.0)	> 3.9	89.3 (73-96)	100.0 (76-100)	<0.0001
	$CD8^+CD27^+$	0.9 (0.8-1.0)	< 54.9	85.2 (67-94)	84.6 (58-97)	<0.0001
	$CD8^+CD28^+$	0.8 (0.7-1.0)	< 38.9	61.1 (39-80)	92.3 (67-100)	0.0031
	$CD4^+CD62L^-$	0.8 (0.6-0.9)	< 31.6	80.0 (61-91)	76.9 (50-92)	0.0033

AUC = Area under the ROC curve; Se = Sensitivity; Sp = Specificity. *Significance was considered at $p<0.05$ and $p<0.01$.

Table S3. Overall performance of cellular phenotypes from COVID-19 patients along the timeline kinetics according to disease outcome

	Disease Outcome - Discharge *vs.* Death					
**Timeline**	**Cell subset**	**AUC**	**Cut-off**	**Se (95% IC)**	**Sp (95% IC)**	***p value***
**D0**	$CD3^+$	0.8 (0.6-0.9)	> 44.7	78.9 (62-89)	73.8 (58-85)	0.0003
**D7**	$CD4^+TIM\text{-}3^+$	1.0 (1.0-1.0)	> 1.9	100.0 (56-100)	100.0 (44-100)	0.0253
	$CD3^+TIM\text{-}3^+$	0.9 (0.7-1.0)	> 3.7	80.0 (37-99)	100.0 (44-100)	0.0526
	$CD4^+CD45RO^+CD27^+$	0.8 (0.6-0.9)	< 25.0	71.4 (45.3)	92.9 (68-100)	0.0082
	$CD3^+$	0.8 (0.6-0.9)	< 51.8	78.6 (52-92)	78.6 (52-92)	0.0149
**D14-28**	$CD4^+$	0.8 (0.5-1.0)	< 27.6	72.7 (43-90)	72.7 (43-90)	0.0386

AUC = Area under the ROC curve; Se = Sensitivity; Sp = Specificity. *Significance was considered at $p<0.05$ and $p<0.01$.
